# Regulatory Architecture of Gene Expression Variation in the Threespine Stickleback *Gasterosteus aculeatus*

**DOI:** 10.1534/g3.116.033241

**Published:** 2016-11-10

**Authors:** Victoria L. Pritchard, Heidi M. Viitaniemi, R. J. Scott McCairns, Juha Merilä, Mikko Nikinmaa, Craig R. Primmer, Erica H. Leder

**Affiliations:** * Division of Genetics and Physiology, Department of Biology, University of Turku, Finland; † Ecological Genetics Research Unit, Department of Biosciences, University of Helsinki, Finland; ‡ Institut Méditerranéen de la Biodiversité et d’Ecologie Marine et Continentale, Marseille, France; § Natural History Museum, University of Oslo, 0318, Norway; ** Institute of Veterinary Medicine and Animal Sciences, Estonian University of Life Sciences, Tartu, Estonia

**Keywords:** Baltic Sea, eQTL, gene expression, liver, threespine stickleback

## Abstract

Much adaptive evolutionary change is underlain by mutational variation in regions of the genome that regulate gene expression rather than in the coding regions of the genes themselves. An understanding of the role of gene expression variation in facilitating local adaptation will be aided by an understanding of underlying regulatory networks. Here, we characterize the genetic architecture of gene expression variation in the threespine stickleback (*Gasterosteus aculeatus*), an important model in the study of adaptive evolution. We collected transcriptomic and genomic data from 60 half-sib families using an expression microarray and genotyping-by-sequencing, and located expression quantitative trait loci (eQTL) underlying the variation in gene expression in liver tissue using an interval mapping approach. We identified eQTL for several thousand expression traits. Expression was influenced by polymorphism in both *cis*- and *trans*-regulatory regions. *Trans*-eQTL clustered into hotspots. We did not identify master transcriptional regulators in hotspot locations: rather, the presence of hotspots may be driven by complex interactions between multiple transcription factors. One observed hotspot colocated with a QTL recently found to underlie salinity tolerance in the threespine stickleback. However, most other observed hotspots did not colocate with regions of the genome known to be involved in adaptive divergence between marine and freshwater habitats.

It is now known that much adaptive evolution is underlain by changes in regions of the genome regulating gene expression, rather than in the protein coding regions of the genes themselves (Pavey *et al.* 2010). Recent work has demonstrated that much variation in gene expression is heritable, and thus evolvable via selection (*e.g.*, Ayroles *et al.* 2009; Powell *et al.* 2013; Leder *et al.* 2015). Correspondingly, studies using model species have found that the genetic polymorphisms underlying phenotypic variation are typically not within genes (Flint and Mackay 2009). Variation in gene expression has been shown to underlie several well-documented cases of phenotypic and/or adaptive divergence. These include plumage coloration and beak shape in birds (Mallarino *et al.* 2011; Poelstra *et al.* 2015), mimetic wing patterns in butterflies (Reed *et al.* 2011; Hines *et al.* 2012), and flower color (Durbin *et al.* 2003). Further, differences in gene expression patterns have been found to correlate with adaptive divergence in multiple species (*e.g.*, Bernatchez *et al.* 2010; Barreto *et al.* 2011). Dysregulation of gene expression due to interactions among regulatory loci has the potential to cause reduced fitness of interpopulation hybrids and thus contribute to reproductive isolation (Ellison and Burton 2008; Turner *et al.* 2014). However, it may also promote hybrid speciation by enabling hybrids to exploit new niches (Lai *et al.* 2006).

The genetic architecture of gene expression regulation can be investigated by treating expression variation as a quantitative trait and identifying the genomic locations associated with it (termed “eQTL”). Such studies have shown that the expression of a gene can be regulated by multiple genomic regions, which are traditionally classified as either *cis* or *trans*. *Cis* regulators, including promoters that activate transcription and enhancers that influence transcription levels, are located close to the regulated gene(s). They contain binding sites for regulatory molecules (proteins or mRNA) that are produced by more distant, *trans* regulators. As *cis* regulators are expected to affect only one or a few focal genes, while *trans* regulators may have pleiotropic effects on many genes, *cis* and *trans* regulators are subject to different evolutionary dynamics. *Cis*-regulatory changes are expected to be important drivers of local adaptation (Wray, 2007), while *trans*-regulatory variation is considered more likely to be under purifying selection (Schaefke *et al.* 2013 but see also Landry *et al.* 2005 for discussion of *cis–trans* coevolution). Correspondingly, *trans*-regulatory polymorphisms tend to affect gene expression less strongly than *cis* polymorphisms, and their effects are more likely to be nonadditive (Zhang *et al.* 2011; Gruber *et al.* 2012; Schaefke *et al.* 2013; Meiklejohn *et al.* 2014; Metzger *et al.* 2016). Nevertheless, work in multiple species has demonstrated an important role for both *cis* and *trans* polymorphism in shaping expression variation (Cubillos *et al.* 2012; Meiklejohn *et al.* 2014; Guerrero *et al.* 2016), and the role of *trans* variation may have been underestimated due to the higher statistical power required to detect it (Mackay *et al.* 2009; Clément-Ziza *et al.* 2014). Interactions involving *trans* regulators may be particularly important in reducing the fitness of interpopulation hybrids (Turner *et al.* 2014). Supporting the pleiotropic role of *trans* regulators, a ubiquitous feature of eQTL studies is the identification of “*trans*-eQTL hotspots,” genomic locations associated with expression variation in many distant genes that are thought to harbor one or more important *trans* regulators (Wu *et al.* 2008; Clément-Ziza *et al.* 2014; Meiklejohn *et al.* 2014).

The threespine stickleback (*Gasterosteus aculeatus*) is an important model in the study of adaptive evolution. Ancestral anadromous populations of threespine stickleback have repeatedly and independently colonized freshwater throughout the Northern Hemisphere (Taylor and McPhail 2000; Mäkinen *et al.* 2006). Sympatric and parapatric freshwater populations may exploit different habitats (Schluter and McPhail 1992; Roesti *et al.* 2012). The species is also distributed throughout semimarine environments with large temperature and salinity gradients, such as estuaries and the brackish water Baltic Sea (McCairns and Bernatchez 2010; Guo *et al.* 2015; Konijnendijk *et al.* 2015). Successful colonization of these diverse habitats necessitates behavioral, morphological, and physiological adaptation to novel environmental conditions including changed temperature, salinity, oxygen, light, parasite and predator regimens, a process that can occur rapidly (Kitano *et al.* 2010; Barrett *et al.* 2011; Terekhanova *et al.* 2014; Lescak *et al.* 2015; Huang *et al.* 2016, Rennison *et al.* 2016). Parallel adaptations between independently founded freshwater populations frequently involve the same regions of the genome and arise from preexisting genetic variation in the marine population (Colosimo *et al.* 2005; Hohenlohe *et al.* 2010; Jones *et al.* 2012; Liu *et al.* 2014; Conte *et al.* 2015, but see DeFaveri *et al.* 2011; Leinonen *et al.* 2012; Ellis *et al.* 2015; Ferchaud and Hansen 2016). Local adaptation in environmentally heterogeneous habitats such as the Baltic Sea (Guo *et al.* 2015) and lake–stream complexes (Roesti *et al.* 2015) has been shown to involve the same genomic regions. Evidence suggests that much of this adaptation may be due to changes in gene regulation rather than protein structure (Jones *et al.* 2012). In addition, plasticity in gene expression in response to different environmental conditions may facilitate initial colonization of novel habitats. (McCairns and Bernatchez 2010; Morris *et al.* 2014). Leder *et al.* (2015) recently demonstrated substantial heritability of expression variation, over thousands of genes, within a Baltic Sea threespine stickleback population, confirming that it can be shaped by local selection. One well-documented locally adaptive trait, reduction of the pelvic girdle, is known to be underlain by variation in the *cis*-regulatory region of the *PITX1* gene (Chan *et al.* 2010), and *cis*-regulatory variation at the *BMP6* gene underlies divergent tooth number between a freshwater and marine population (Cleves *et al.* 2014). Differences in levels of thyroid hormone between freshwater and marine sticklebacks, which are connected to different metabolic rates between the two environments, are associated with *cis*-regulatory variation at the *TSH*β*2* gene (Kitano *et al.* 2010). Recently, Di Poï *et al.* (2016) showed that differences in behavior and response to stress between marine and freshwater sticklebacks may be modulated by variation in the expression of hormone receptors. Otherwise, the architecture of gene expression regulation in the threespine stickleback and its role in adaptive evolution is only starting to be explored (Chaturvedi *et al.* 2014).

Understanding the regulatory pathways underlying variation in gene expression, and how this gene expression variation influences the phenotype, will improve our understanding of how organisms can adapt to novel environments and, thus, how adaptive diversity is generated. In the stickleback, for example, it is unknown whether regulatory loci involved in local adaptation are clustered on the regions of the genome that show repeated divergence in independent marine-freshwater colonizations. Here, we perform the first genome-wide study of this regulatory architecture in the threespine stickleback, by mapping QTL underlying the variation in expression of several thousand genes in a population from the Baltic Sea. We examine transcription in the liver, a metabolically active tissue that expresses many genes potentially involved in physiological adaptation to different aquatic habitats.

## Materials and Methods

### Experimental crosses

We used a multi-family, paternal half-sib cross design for QTL mapping. Crossing procedures have previously been detailed in Leinonen *et al.* (2011) and Leder *et al.* (2015). In short, 30 mature males and 60 gravid females were collected from the Baltic Sea for use as parents. Each male was artificially crossed with two females, producing 30 half-sib blocks each containing two full-sib families. Families were reared in separate 10 L tanks with density standardized to 15 individuals per tank, temperature at 17 ± 1°, and 12:12 hr light/dark photoperiod. At the age of 6 months, 10 offspring from each family (five treated and five controls) were subjected to a temperature treatment as part of a related experiment (control: constant 17°; treatment: water gradually heated from 17 to 23° over 6 hr, see Leder *et al.* 2015), and immediately killed for DNA and RNA collection.

### RNA preparation, microarray design, and data normalization

RNA preparation, gene expression microarrays, hybridization, and normalization procedures are described in detail in Leder *et al.* (2009, 2015). Briefly, total RNA was isolated from offspring liver tissue using standard protocols. RNA that passed quality thresholds was labeled (Cy3 or Cy5) using the Agilent QuickAmp Kit, with equal numbers of individuals within family groups (control and temperature-treated; males and females) assigned to each dye. Labeled RNA was hybridized to a custom 8 × 15K microarray, with sample order randomized (Agilent Hi-RPM kit). Labeling, hybridization, and scanning was performed at the University Health Network in Toronto, Canada. Images of the arrays were acquired, image analysis was performed, and array quality was assessed as detailed in Leder *et al.* (2015). Postprocessed signals were standardized across arrays using a supervised normalization approach, implemented in the package “snm” for R/Bioconductor (Mecham *et al.* 2010; R Core Team 2015). Dye, array, and batch (*i.e.*, slide) were defined as “adjustment variables”; sex, family, and temperature treatment were defined as “biological variables.” Following normalization, individual intensity values more than two SD from their family-by-treatment mean, and probes with missing values for an entire family or > 10% of individuals were removed. The final dataset contained 10,527 expression traits (10,495 genes plus 32 additional splice variants) and 563 individuals (158 control females, 125 control males, 152 treated females, and 128 treated males).

### Genotyping-by-sequencing

For genotyping-by-sequencing of parents (*n* = 90) and offspring (*n* = 580), we used the method of Elshire *et al.* (2011) with an additional gel excision step to improve size selection. DNA was extracted from ethanol-preserved fin tissue (parents) or frozen liver tissue (offspring), and DNA concentrations were measured using a NanoDrop ND-1000 spectrophotometer. DNA (80 ng) was digested with the restriction enzyme *Pst*1 1.5 U (New England Biolabs) and 1 × NEB buffer 3, 1 × bovine serum albumin (BSA), and dH_2_O (3.3 µl) in a thermocycler (37°, 2 hr; 75°, 15 min; and 4°, 10 min). The digested DNA was ligated to adapters with T4-ligase 0.6 × (New England Biolabs), 1 × Ligase Buffer, 21 µl dH_2_O, and 50 nM of pooled forward and reverse adapters, which were prepared according to Elshire *et al.* (2011) (ligation program: 22°, 1 hr; 65°, 30 min; and 4°, 10 min). Up to 104 unique barcodes were used in each library to label individual samples. The ligation products were pooled into libraries and purified with a QIAquick PCR Purification Kit (QIAGEN). The purified libraries were PCR amplified with the following components: purified ligated library (20 µl), reaction buffer 1 ×, MgCl_2_ 1.5 nM (Bioline), primer mix 0.5 µM, dNTPs (Fermentas) 0.4 μM, BioTaq 0.05 U (Bioline), and dH_2_O (20 µl) [amplification program: 72°, 5 min; 4 cycles (95°, 30 sec; 95°, 10 sec; 65°, 30 sec; and 70°, 30 sec); 11 cycles (95°, 10 sec; 65°, 30 sec; and 72°, 20 sec); 72°, 5 min; and 4°, 10 min]. Lastly, we performed a manual size selection by loading 40 µl of the amplified library on a gel [MetaPhor (Lonza) 2.5%, 150 ml, and 100 V for 1.5 hr] and cutting the 300–400 bp range from the resultant smear. The DNA was extracted from the gel with a QIAquick Gel Extraction Kit (QIAGEN). The cleaned product was again separated on a gel, cut, and cleaned.

All products were sequenced with paired-end reading on the Illumina HiSeq2000 platform. Six hundred and fifty individuals, multiplexed into 10 separate libraries (maximum library size = 104 individuals), were sequenced at the Beijing Genomics Institute; 55 individuals (including duplicates) were sequenced at the Finnish Institute for Molecular Medicine or at the University of Oslo.

### Variant calling

Reads were split by barcode, and barcodes removed, using a custom perl script. Low-quality bases were removed from the reads via window adaptive trimming using Trim.pl (available: https://github.com/LJI-Bioinformatics/HLATyphon/blob/master/01.Pre_Processing/trim.pl, Illumina quality score ≤ 20). Paired-end reads for each of these individuals were aligned to the BROAD S1 stickleback genome using BWA aln/sampe (v 0.6.2) with default parameters (Li and Durbin 2009). The threespine stickleback genome comprises 21 assembled chromosomes plus 1823 unplaced genomic scaffolds. Unmapped reads, and reads with nonunique optimal alignments, pair-rescued alignments, or any alternative suboptimal alignments, were discarded from the resulting SAM files. SAM files were converted to sorted BAM files using samtools 0.1.18 (Li *et al.* 2009) and variants were called within each paternal family using the samtools mpileup function with extended BAQ computation (options: -AED, max-depth 500), in combination with bcftools (Li *et al.* 2009). We did not degrade mapping quality for reads with large numbers of mismatches as we found this to reject high-quality reads due to fixed polymorphisms between our European stickleback samples and the North American stickleback genome. Indel and multi-allelic variants were discarded. Initial filters based on SNP quality and variability within and across families resulted in a list of 26,290 candidate biallelic SNPs for further analysis. Samtools and bcftools, applied to each paternal family separately, were then used to call each individual for the genotype at each of the 26,290 sites. Sites at which bcftools identified multiple variant types (SNPs, indels, and multi-base polymorphisms) within and among families were removed, leaving 25,668 successfully genotyped variant sites.

### Genotype quality control

Vcftools (Danecek *et al.* 2011) was used to recode genotypes with a genotype quality phred score (GQ) < 25 or a sequencing depth (DP) < 8 or > 1000 to missing. Vcf files for all families were merged and the merged file converted to the input format for Plink 1.07 (Purcell *et al.* 2007). For SNPs on all autosomal chromosomes and the pseudoautosomal region of chromosome 19 (see below), the following filters were applied in Plink: hwe (based on founders only) < 0.01, maximum missing genotypes = 0.25, minor allele frequency > 0.05, and offspring with > 70% missing data removed. Adjacent SNPs in complete linkage disequilibrium were manually consolidated into a single locus, with combined SNP information used to call genotypes.

Several approaches were used check for sample contamination or errors in barcode splitting and family assignment: in Plink, the *mendel* option was used to screen families for Mendelian errors, and sample relatedness was examined by graphically visualizing genome-wide IBD-sharing coefficients generated by *genome*; the program SNPPIT (Anderson 2012) was used to assign individuals to parents, based on five independent datasets of 100 SNPs; and 220 SNPs on Stratum II of chromosome 19 (see below) were examined for their expected pattern in males and females (all heterozygous in males *vs.* all homozygous in females).

The stickleback chromosome 19 is a proto-sex chromosome (Peichel *et al.* 2004; Roesti *et al.* 2013; Schultheiß *et al.* 2015), with a normally recombining pseudoautosomal domain (∼0–2.5 Mb), a nonrecombining domain in the male version (Stratum I, ∼2.5–12 Mb), and a domain largely absent in the male version (Stratum II, ∼12–20 Mb). For Stratum I, parental and offspring genotypes were inspected manually in order to identify the male-specific allele and this was recoded to a unique allele code (“9”) for the purposes of linkage map construction. Where the male-specific allele could not be identified, all genotypes within a family were recoded as missing. Genotypes were also inspected manually for Stratum II, and any SNP found to be heterozygous in males was excluded. All remaining Stratum II SNPs were considered to be hemizygous in males, and one of the alleles was also recoded as “9.”

### Linkage map construction

We constructed a linkage map using the improved version of Crimap (Green *et al.* 1990, available: http://www.animalgenome.org/tools/share/crimap/). Remaining Mendelian errors in the dataset were removed using the *set-me-missing* option in Plink. For each SNP, the number of informative meioses were examined using Crimap, and markers with < 150 informative meioses or within 500 bp of one another were discarded.

The initial map build included 6448 markers. Where applicable, SNPs were ordered according to the modified genome build of Roesti *et al.* (2013). We attempted to position all previously unplaced scaffolds containing at least two genotyped SNPs on to the map. Scaffolds were assigned to chromosome on the basis of LOD score using the Crimap function *two-point*, and then positioned using a combination of information from pilot Crimap *builds*, *chrompic*, and *fixed* together with known start and end points of previously assembled scaffolds (Roesti *et al.* 2013). Information from *chrompic* and *fixed* were also used to confirm the orientation of scaffolds newly placed by Roesti *et al.* (2013). Once all possible scaffolds had been placed, recombination distance between ordered SNPs was estimated using *fixed*. To refine the map, we iteratively removed SNP genotypes contributing to implied double crossovers within a 10 cM interval (presumed to be genotyping errors) and SNPs generating recombination distances of > 1 cM per 10,000 bp, and recalculated distances using *fixed*. Remaining regions of unusually high recombination on the map were investigated by examining whether removal of individual SNPs altered map distance.

### eQTL identification

eQTL were identified using an interval mapping approach (Knott *et al.* 1996) implemented in QTLMap 0.9.0 (http://www.inra.fr/qtlmap; QTLMap option: *–data-transcriptomic*). Offspring with missing genotypes at > 60% of the markers in the linkage map were removed from the analysis. We applied linkage analysis assuming a Gaussian trait distribution (QTLMap option: *–calcul = 3*), and included dye, temperature treatment, and sex as fixed factors in the model. Due to the relatively small size of some of our half-sib families, we examined sire effects only, with a separate QTL effect estimated for each sire. Excluding dam effects is expected to reduce our power of eQTL detection, as fewer parents will be segregating for each QTL.

A fast algorithm was used to identify phase and estimate transmission probabilities at each chromosomal location (Elsen *et al.* 1999, QTLMap option: *–snp*). Autosomes and the pseudoautosomal portion of the sex chromosome were scanned at 1 cM intervals, and the presence of QTL on a chromosome was assessed using a likelihood ratio test (LRT) under the hypothesis of one *vs.* no QTL. Chromosome-wide LRT significance thresholds for each trait were identified empirically, by permuting fixed effects and traits among individuals within families and recalculating LRT scores (5000 permutations). As the combination of 5000 permutations × 10,332 traits × 21 chromosomes was computationally prohibitive, we first performed permutations on a subset of 200 expression traits to establish a LRT threshold below which identified QTL were unlikely to be significant at chromosome-wide *P* < 0.05 (LRT = 55), and then used permutations to assess the significance of all QTL above this threshold. The nonpseudoautosomal region of the female chromosome 19 can be considered analogous to the X chromosome; identification of QTL in this region requires estimation of dam effects and was therefore not performed. The 95% C.I. for each QTL was estimated using the drop-off method implemented in QTLMap 0.9.7, which returns flanking map positions plus their nearest marker.

### Cis- *vs.* trans-eQTL

To discriminate *cis*- *vs.* *trans*-QTL, we compared inferred QTL location to the position of the expressed gene according to the BROAD *G. aculeatus* genome annotation v. 1.77 (available at http://ftp.ensembl.org/pub/release-77/gtf/gasterosteus_aculeatus/). All positions on the BROAD annotation were recoded to positions on our modified chromosome assemblies. For genes on scaffolds un-anchored to our assembly, we also used information on chromosomal scaffold locations available in the recently published map of Glazer *et al.* (2015). Any eQTL on a different chromosome from the regulated gene was considered *trans*. For eQTL on the same chromosome as the gene, we initially considered two alternative threshold distances for an eQTL to be considered *trans* [> 1 Mb following Grundberg *et al.* (2012) or > 10 Mb following van Nas *et al.* (2010)]. For the 1 Mb threshold, we observed strong enrichment of significant *trans*-eQTL on the same chromosome as the regulated gene, indicating that these were actually mis-identified *cis*-eQTL; therefore, we selected the conservative 10 Mb threshold. In practice, examination of our results showed that 95% C.I. of eQTL sometimes extended further than this 10 Mb threshold. Considering median 95% C.I. (∼1 Mb), we therefore classified a QTL as *trans* if the SNP closest to the upper or lower 95% confidence bounds of that QTL was further than 9.5 Mb from the regulated gene. Following Johnsson *et al.* (2015), we applied a local significance threshold (chromosome-wide *P* < 0.01) for evaluation of possible *cis*-QTL and a genome-wide significance threshold (genome-wide *P* < 0.021, = chromosome-wide threshold of 0.001 * 21 chromosomes) for evaluation of possible *trans*-QTL. Although this significance threshold is permissive, we considered it acceptable as our aim was to analyze the eQTL distribution across the genome rather than to identify individual QTL-locus associations. Similar significance thresholds have been used for eQTL detection in comparable studies (*e.g.*, Whiteley *et al.* 2008).

To ask whether the effect of variation in *trans*-regulatory sites was more often nonadditive than the effect of variation in *cis*-regulatory sites, we examined the narrow sense heritability (*h*^2^) and dominance proportion of genetic variance (*d*^2^) estimated for each expression trait by Leder *et al.* (2015) and provided in the Supplemental Data for that paper.

### Genes with plastic *vs.* nonplastic expression

To investigate whether genes exhibiting an alteration in expression level in response to a temperature stress treatment (*i.e.*, those exhibiting environmental plasticity) had a different underlying regulatory architecture to those not exhibiting such a response, we divided genes into a “responding” and “nonresponding” group based on the results provided in the Supplementary Data for Leder *et al.* (2015) and compared the frequency and position of *cis*- and *trans*-eQTL between the two groups.

### Evaluation of eQTL hotspots

As all identified eQTL had a wide 95% C.I., meaning that physically close eQTL positions could be due to the effect of the same locus (see below), we evaluated potential eQTL hotspots by counting eQTL within 5 cM bins across the genome (“hotspot size” = number of eQTL). Where the number of 1 cM bins within a chromosome was not a simple multiple of five, bin sizes at the start and/or end of the chromosome were increased to six or seven. To obtain an empirical significance threshold above which clusters of eQTL could be considered a “hotspot,” we simulated the expected neutral distribution of eQTL across the genome using a custom script. We performed 5000 simulations: for each, we assigned *n* eQTL (where *n* = relevant number of significant eQTL) randomly across the 3062 1 cM bins of the genome and then summed them into 5 cM (or larger) bins as described above. Conservatively, we compared the size of hotspots in the real data to the size distribution of the largest hotspot observed over each of the 5000 simulations.

### Association of eQTL with regions under selection

Hohenlohe *et al.* (2010), Jones *et al.* (2012), and Terekhanova *et al.* (2014) documented parallel regions of the genome divergent between marine and freshwater sticklebacks on chromosomes 1, 4 (three regions), 7, 11, and 21. We investigated whether these regions harbored important *trans* regulators that might contribute to adaptation to different aquatic habitats by comparing the location of these regions with the location of our identified *trans*-eQTL hotspots. We also compared hotspot locations to regions of the genome inferred by Guo *et al.* (2015) to be involved in adaptive differentiation among different stickleback populations in the Baltic Sea.

### Ortholog identification

In order to maximize the functional information available, we identified human orthologs for *G. aculeatus* genes. As a first attempt, we used BioMart (Durinck *et al.* 2005; Smedley *et al.* 2009) to identify human orthologs and obtain the HGNC symbols for the human genes. When BioMart failed to return a human ortholog, protein BLAST searches were used to identify orthologs using the Ensembl human protein database. The identifier conversion tool, db2db, from bioDBnet (https://biodbnet-abcc.ncifcrf.gov/db/db2db.php) was used to convert between Ensembl identifiers and HGNC gene symbols when needed (Mudunuri *et al.* 2009).

### Hotspot annotation

To identify regulatory genes physically associated with an eQTL hotspot, we defined hotspot confidence boundaries as being the most frequently observed 95% confidence limits of all significant eQTL centered in the hotspot. We used AmiGO2 (Carbon *et al.* 2009) to identify “molecular function” or “biological process” Gene Ontology (GO) terms associated with transcriptional regulation by applying the search term “transcription regulation and – pathway.” We then used BioMart to examine all genes within the hotspot boundaries for any of these GO annotations, using the HGNC symbols as input. As an important transcriptional regulator generating a hotspot might itself be regulated by the hotspot rather than physically present within it, we repeated this analysis for all genes with eQTL mapped to the hotspot (*cis*-eQTL significant at chromosome-wide *P* < 0.01; *trans*-eQTL significant at genome-wide *P* < 0.021). We used DAVID (Huang *et al.* 2009a,b) to examine GO term enrichment for the sets of genes with *trans*-QTL mapping to each hotspot, using the 9071 genes on the microarray with identified human orthologs as the background. To increase our sample size, we lowered our stringency and examined all genes with *trans*-eQTL mapping to the hotspot locations at genome-wide *P* < 0.057 (chromosome-wide *P* < 0.0027).

### Upstream regulator and functional interaction analyses

To search for regulatory genes that may be responsible for the expression variation in genes with identified *trans*-eQTL, we used the upstream regulator analysis in the Ingenuity Pathway Analysis (IPA) software (QIAGEN). This analysis uses a Fisher’s Exact Test to determine whether genes in a test dataset are enriched for known targets of a specific transcription factor. We used the human HGNC symbols as identifiers in IPA. First, we examined all genes that had a significant *trans*-eQTL mapping to any location at a genome-wide *P* < 0.021 (chromosome-wide *P* < 0.001). To investigate the upstream regulators potentially involved in generating eQTL hotspots in more detail, we lowered our stringency and also examined all genes with *trans*-eQTL mapping to the hotspot locations at genome-wide *P* < 0.057 (chromosome-wide *P* < 0.0027).

Since transcription is typically initiated by a complex of genes rather than a single transcription factor, we examined functional relationships among the identified upstream regulators for each hotspot (Supplemental Material, Table S8), the genes located within a hotspot, and the genes with significant eQTL mapping to that hotspot (Table S4; *cis*-eQTL significant at chromosome-wide *P* < 0.01, *trans*-eQTL significant at genome-wide *P* < 0.021), using STRING v10 (Jensen *et al.* 2009, http://string-db.org/). We searched for evidence of functional relationships from experiments, databases, and gene coexpression, and applied a minimum required interaction score of 0.4.

### Data availability

QTLMap input files are provided as Files Files S1-S5. Raw and normalized microarray data, in addition to R scripts describing the normalization procedure, are available in the ArrayExpress database (www.ebi.ac.uk/arrayexpress) under accession number E-MTAB-3098. RAD sequence reads for each individual have been deposited in the NCBI Sequence Read Archive under BioProject ID PRJNA340327. Further information about archived data is provided in File S6.

## Results

### Genotyping-by-sequencing

Sufficient numbers of reads were obtained for 620 of the 670 individuals sent for sequencing. Fifteen of these individuals failed initial quality control steps. For the 605 sticklebacks (88 parents and 517 offspring) that were retained for analysis, we obtained a total of 583,032,024 raw paired reads (40,357–11,940,726 per individual, median = 834,286). Approximately 67% of these reads remained aligned to the stickleback genome following removal of reads with nonunique optimal alignments, any alternative suboptimal alignments, or pair-rescued alignments (range 36.2–78.8%, median = 70.1%). Raw read and alignment statistics for each individual are provided in Table S1.

### Linkage map construction

**Figure 1:**
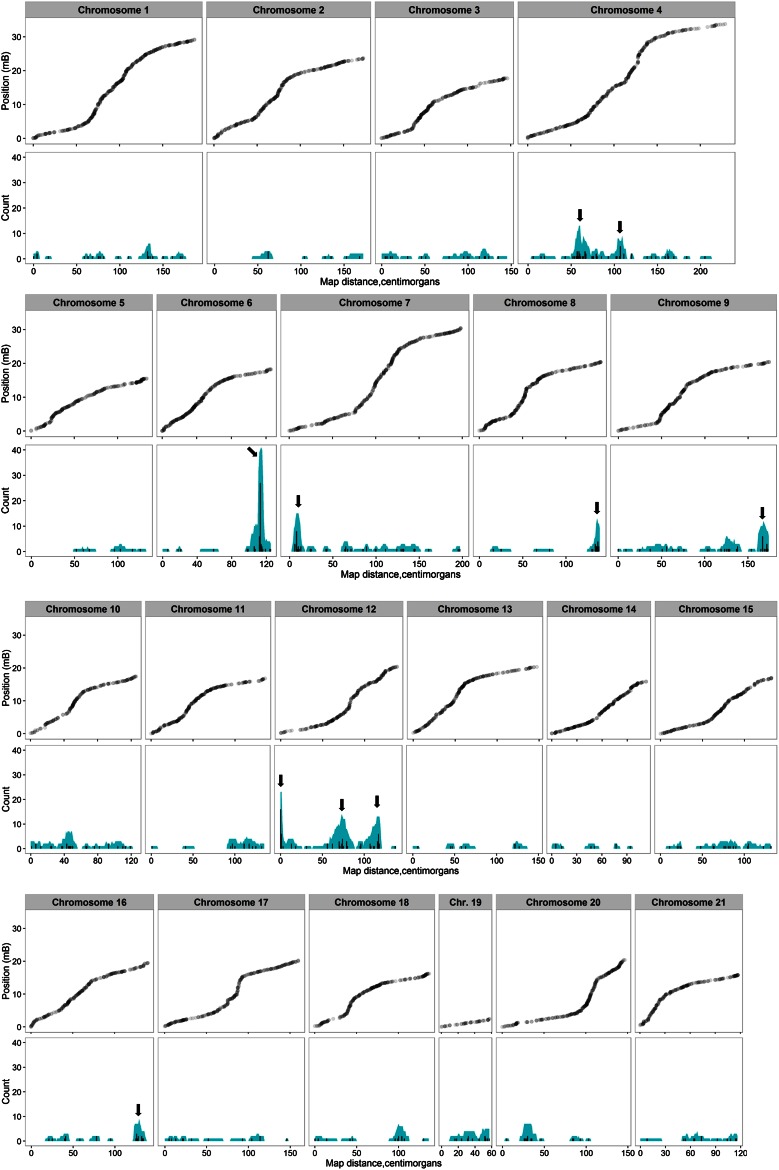
Position of SNP markers along each chromosome (top) and location of *trans*-eQTL hits for all assayed genes (bottom). Black bars show the number of eQTL hits at each 1 cM Kosambi interval along the chromosome. Blue shading shows the number of eQTL with 95% C.I. overlapping each 1 cM interval. Arrows indicate the location of ten significant *trans* eQTL hotspots. Figure created using ggplot2 (Wickham 2009) in R. eQTL, expression quantitative trait loci; SNP, single nucleotide polymorphism.

Following SNP calling and quality control steps, 13,809 of the original 25,668 SNPs, genotyped in 605 individuals (mean number of offspring per family = 18), were available for linkage map construction. Following removal of markers with < 150 informative meioses or within 500 bp, 6448 SNPs were included in the initial map build. The final sex-averaged linkage map spanned 3110 cM Kosambi (including the complete chromosome 19) and included 5975 markers, of which ∼45% were located at the same map position as another marker (Figure 1, Figure S1, and Table S2). Forty-three previously un-placed scaffolds (10.35 Mb) were added to the chromosome assemblies of Roesti *et al.* (2012) (Table S3). Thirty-five of these scaffolds were also recently added to the stickleback assembly in an independent study by Glazer *et al.* (2015). Although there were some differences in scaffold orientation, location of the new scaffolds was almost completely congruent between the two maps (Table S3). For QTL detection with QTLMap, the map was reduced to 3189 SNPs with unique positions (average intermarker distance = 0.98 cM, Table S2).

### Identification of cis- and trans-eQTL

Expression data were available for 500 of the 517 genotyped offspring. Twenty-six of these offspring had > 60% missing genotype data and were removed from the analysis. As we found that missing values in the expression trait file caused QTLMap to overestimate the LRT statistic, we eliminated these from the dataset by removing one additional individual and 195 expression traits. Eighty-eight genotyped parents, 473 genotyped and phenotyped offspring (mean no. offspring per family = 15.8, mean proportion of missing genotypes in offspring = 0.11; maximum = 0.56), and 10,332 expression traits were retained for the analysis. At chromosome-wide *P* < 0.01, we identified 5366 eQTL associated with 4507 expression traits (43.7% of the 10,322 expression traits examined, Table S4). Based on our recoded gene positions, we classified 2335 of these as *cis*-eQTL, 2870 as *trans*-eQTL, and 161 as unknown; that is, the expressed gene was located on a scaffold that had not been assigned to a *G. aculeatus* chromosome by either this study or Glazer *et al.* (2015) (Table S4, and Table S5). Four hundred and seventy-four of the *trans*-eQTL were significant at genome-wide *P* < 0.021. Of these, 84.5% mapped to a chromosome other than the one containing the regulated gene. After application of this genome-wide significance threshold for *trans*-eQTL, 2858 expression traits (27.7% of those examined) remained associated with one or more significant *cis*- or *trans*-eQTL. Of these, 79.4% were associated with a *cis*-eQTL, 13.9% with one or more *trans*-eQTL, 2.3% with both a *cis*- and a *trans*-eQTL, and 4.4% with eQTL of unknown class (Table S4). The physical distribution across the genome of the 2858 loci with significant *cis*- or *trans*-eQTL is shown in Figure S2. Mean 95% C.I. of significant eQTL was 10.2 cM (range 1–86 cM), ∼1.77 Mb (range 0.03–22.19 Mb). Overall, *trans*-regulated expression traits did not exhibit more dominance variance than *cis*-regulated loci (*trans*-regulated loci, mean *h*^2^ = 0.31, mean *d*^2^ = 0.16; *cis*-regulated loci: mean *h*^2^ = 0.37, mean *d*^2^ = 0.18; values from Leder *et al.* 2015).

### Trans-eQTL hotspots

*Trans*-eQTL (significant at genome-wide *P* < 0.021) were not evenly distributed across the genome and we identified ten 5 cM bins, located on seven different chromosomes, as containing eQTL clusters (seven or more eQTL; *P* < 0.012 based on the largest hotspot observed in neutral simulations; Figure 1). A particularly large eQTL hotspot (36 *trans*-eQTL within the 5 cM bin) was identified close to one end of chromosome 6, three hotspots (23, 7, and 8 *trans*-eQTL) were present at separate locations on chromosome 12, two hotspots (10 and 7 *trans*-eQTL) were located on chromosome 4, and the remaining hotspots were located near the ends of chromosomes 7, 8, 9, and 16 (14, 10, 8, and 7 *trans*-eQTL). To eliminate the possibility that distant *cis*-eQTL misclassified as *trans* were contributing to observed hotspots, we repeated the analysis with the 401 *trans*-eQTL that were on a different chromosome to their regulatory target; 9 out of the 10 hotspots were still present (six or more eQTL, *P* < 0.038; second chromosome 4 hotspot, with five eQTL, no longer significant). Physical hotspot boundaries were assigned from inspection of eQTL hits and 95% C.I. as follows: chromosome 4, 55–67 cM (“Chr4a,” 4,630,680–6,394,113 bp); chromosome 4, 104–113 cM (“Chr4b,” 15,643,256–17,021,069 bp), chromosome 6, 111–116 cM (“Chr6,” 17,238,934–17,469,219 bp); chromosome 7, 5–12 cM (“Chr7,” 396,541–1,107,393 bp); chromosome 8, 134–139 cM (“Chr8,” 19,917,746–20,316,565 bp); chromosome 9, 165–174 cM (“Chr9,” 19,822,078–20,440,410 bp); chromosome 12, 0–1 cM (“Chr12a,” 0–337,849 bp); chromosome 12, 72–79 cM (“Chr12b,” 5,853,981–7,440,742 bp); chromosome 12, 109–119 cM (“Chr12c,” 15,551,555–17,229,387 bp); and chromosome 16, 123–130 cM (“Chr16,” 17,658,526–18,257,571 bp).

### Genes with plastic *vs.* nonplastic expression

Following FDR correction, 4253 genes were found by Leder *et al.* (2015) to exhibit a significant change in expression in response to a temperature treatment. We identified significant eQTL underlying 1131 of these genes (Table S4; eQTL type: 79.8% *cis*, 12.7% *trans*, 2.6% both, and 4.9% unknown). The distribution of the 177 significant *trans*-eQTL across 5 cM bins indicated four hotspots (five or more eQTL, *P* < 0.01, Figure S3), all of which had been previously observed in the full dataset. The chromosome 16 hotspot was greatly increased in relative importance (Chr4b: 6 eQTL; Chr 6: 12 eQTL; Chr12a: 9 eQTL; and Chr16: 7 eQTL).

### Association of eQTL with regions under selection

None of our identified eQTL hotspots overlapped parallel regions of the genome divergent between marine and freshwater sticklebacks identified by Hohenlohe *et al.* (2010), Jones *et al.* (2012), and Terekhanova *et al.* (2014), or with the clusters of morphological QTL on chromosome 20 (Miller *et al.* 2014, Table S6). However, one genomic region identified as divergent between marine and freshwater populations by Terekhanova *et al.* (2014) alone overlapped with the Chr12b eQTL hotspot. Only 9 of the 297 genes inferred by Guo *et al.* (2015) as being under selection among Baltic Sea populations experiencing different temperature and salinity regimens overlapped observed eQTL hotspots (Chr4a, Chr4b, Chr7, Chr9, and Chr12b, Table S6).

### Hotspot annotation

We identified human orthologs for 16,315 of the 20,787 protein-coding genes annotated on the Broad stickleback genome (78.5%, Table S5). There were 393 genes with human annotation physically located within the designated boundaries of the eleven hotspots (Table S6). Of these, 70 (17.8%) had a GO term related to transcription regulation (Table 1 and Table S7). In addition, 21 genes with significant *cis*-eQTL or *trans*-eQTL mapping to a hotspot had GO terms related to transcriptional regulation (Table 1 and Table S7). Following correction for multiple testing, we found no significant GO term enrichment among any group of genes *trans*-regulated by the same eQTL hotspot.

**Table 1:** Known transcriptional regulators associated with identified eQTL hotspots

Hotspot	Location	Stickleback Ensembl_ID	Human Ensembl_ID	Gene Name	Description
Chr04a	*Cis*	ENSGACG00000017632	ENSG00000133884	DPF2	Double PHD fingers 2
Chr04a	*Cis*	ENSGACG00000017819	ENSG00000156603	MED19	Mediator complex subunit 19
Chr04a	*Cis*	ENSGACG00000017706	ENSG00000168002	POLR2G	Polymerase (RNA) II subunit G
Chr04a	*Cis*	ENSGACG00000017981	ENSG00000155827	RNF20	Ring finger protein 20
Chr04a	Hotspot	ENSGACG00000017062	ENSG00000175602	CCDC85B	Coiled-coil domain containing 85B
Chr04a	Hotspot	ENSGACG00000017113	ENSG00000131264	CDX4	Caudal type homeobox 4
Chr04a	Hotspot	ENSGACG00000016877	ENSG00000145214	DGKQ	Diacylglycerol kinase θ
Chr04a	Hotspot	ENSGACG00000016862	ENSG00000088881	EBF4	Early B-cell factor 4
Chr04a	Hotspot	ENSGACG00000016923	ENSG00000126500	FLRT1	Fibronectin leucine rich transmembrane protein 1
Chr04a	Hotspot	ENSGACG00000017059	ENSG00000175592	FOSL1	FOS like 1, AP-1 transcription factor subunit
Chr04a	Hotspot	ENSGACG00000017029	ENSG00000184481	FOXO4	Forkhead box O4
Chr04a	Hotspot	ENSGACG00000016896	ENSG00000161021	MAML1	Mastermind like transcriptional coactivator 1
Chr04a	Hotspot	ENSGACG00000016876	ENSG00000109320	NFKB1	Nuclear factor κ B subunit 1
Chr04a	Hotspot	ENSGACG00000017076	ENSG00000174576	NPAS4	Neuronal PAS domain protein 4
Chr04a	Hotspot	ENSGACG00000017018	ENSG00000123728	RAP2C	RAP2C, member of RAS oncogene family
Chr04a	Hotspot	ENSGACG00000017237	ENSG00000147274	RBMX	RNA binding motif protein, X-linked
Chr04a	Hotspot	ENSGACG00000017181	ENSG00000134595	SOX3	SRY-box 3
Chr04a	Hotspot	ENSGACG00000016868	ENSG00000131508	UBE2D2	Ubiquitin conjugating enzyme E2 D2
Chr04a	Hotspot	ENSGACG00000016930	ENSG00000185670	ZBTB3	Zinc finger and BTB domain containing 3
Chr04a	Hotspot	ENSGACG00000017211	ENSG00000152977	ZIC1	Zic family member 1
Chr04a	Hotspot	ENSGACG00000017212	ENSG00000156925	ZIC3	Zic family member 3
Chr04a	*Trans*	ENSGACG00000019192	ENSG00000105856	HBP1	HMG-box transcription factor 1
Chr04a	*Trans*	ENSGACG00000018763	ENSG00000168310	IRF2	Interferon regulatory factor 2
Chr04a	*Trans*	ENSGACG00000010116	ENSG00000163904	SENP2	SUMO1/sentrin/SMT3 specific peptidase 2
Chr04a	*Trans*	ENSGACG00000019776	ENSG00000234495	TRIM27	Tripartite motif containing 27
Chr04b	Hotspot	ENSGACG00000018659	ENSG00000112983	BRD8	Bromodomain containing 8
Chr04b	Hotspot	ENSGACG00000018605	ENSG00000198791	CNOT7	CCR4-NOT transcription complex subunit 7
Chr04b	Hotspot	ENSGACG00000018730	ENSG00000170619	COMMD5	COMM domain containing 5
Chr04b	Hotspot	ENSGACG00000018655	ENSG00000147257	GPC3	Glypican 3
Chr04b	Hotspot	ENSGACG00000018752	ENSG00000171720	HDAC3	Histone deacetylase 3
Chr04b	Hotspot	ENSGACG00000018614	ENSG00000179111	HES7	Hes family bHLH transcription factor 7
Chr04b	Hotspot	ENSGACG00000018626	ENSG00000101928	MOSPD1	Motile sperm domain containing 1
Chr04b	Hotspot	ENSGACG00000018642	ENSG00000156531	PHF6	PHD finger protein 6
Chr04b	Hotspot	ENSGACG00000018664	ENSG00000138814	PPP3CA	Protein phosphatase 3 catalytic subunit α
Chr04b	Hotspot	ENSGACG00000018680	ENSG00000185129	PURA	Purine rich element binding protein A
Chr04b	Hotspot	ENSGACG00000018663	ENSG00000184584	TMEM173	Transmembrane protein 173
Chr04b	*Trans*	ENSGACG00000018210	ENSG00000121060	TRIM25	Tripartite motif containing 25
Chr04b	*Trans*	ENSGACG00000001351	ENSG00000116830	TTF2	Transcription termination factor 2
Chr06	*Cis*	ENSGACG00000012317	ENSG00000266412	NCOA4	Nuclear receptor coactivator 4
Chr06	*Cis*	ENSGACG00000001371	ENSG00000167380	ZNF226	Zinc finger protein 226
Chr06	Hotspot	ENSGACG00000011981	ENSG00000197223	C1D	C1D nuclear receptor corepressor
Chr06	*Trans*	ENSGACG00000018659	ENSG00000112983	BRD8	Bromodomain containing 8
Chr06	*Trans*	ENSGACG00000005983	ENSG00000168036	CTNNB1	Catenin (cadherin-associated protein), β 1, 88 kDa
Chr06	*Trans*	ENSGACG00000004982	ENSG00000065883	CDK13	Cyclin-dependent kinase 13
Chr06	*Trans*	ENSGACG00000008525	ENSG00000100644	HIF1A	Hypoxia inducible factor 1, α subunit
Chr06	*Trans*	ENSGACG00000013704	ENSG00000096968	JAK2	Janus kinase 2
Chr06	*Trans*	ENSGACG00000009631	ENSG00000107938	EDRF1	Erythroid differentiation regulatory factor 1
Chr06	*Trans*	ENSGACG00000018816	ENSG00000196670	ZFP62	ZFP62 zinc finger protein
Chr07	*Cis*/hotspot	ENSGACG00000018669	ENSG00000137462	TLR2	Toll-like receptor 2
Chr07	Hotspot	ENSGACG00000000325	ENSG00000135625	EGR4	Early growth response 4
Chr07	Hotspot	ENSGACG00000018606	ENSG00000109670	FBXW7	F-box And WD repeat domain containing 7, E3 ubiquitin protein ligase
Chr07	Hotspot	ENSGACG00000000304	ENSG00000170448	NFXL1	Nuclear transcription factor, X-box binding-like 1
Chr07	Hotspot	ENSGACG00000000370	ENSG00000164985	PSIP1	PC4 and SFRS1 interacting protein 1
Chr07	Hotspot	ENSGACG00000018586	ENSG00000074966	TXK	Tyrosine kinase
Chr07	*Trans*	ENSGACG00000000333	ENSG00000173801	JUP	Junction plakoglobin
Chr08	Hotspot	ENSGACG00000014457	ENSG00000162733	DDR2	Discoidin domain receptor tyrosine kinase 2
Chr08	Hotspot	ENSGACG00000014404	ENSG00000187764	SEMA4D	Sema domain, immunoglobulin domain (Ig), transmembrane domain (TM) and short cytoplasmic domain, (Semaphorin) 4D
Chr08	Hotspot	ENSGACG00000014374	ENSG00000178078	STAP2	Signal transducing adaptor family member 2
Chr08	*Trans*	ENSGACG00000006033	ENSG00000125686	MED1	Mediator complex subunit 1
Chr08	*Trans*	ENSGACG00000017475	ENSG00000137699	TRIM29	Tripartite motif containing 29
Chr08	*Trans*	ENSGACG00000003512	ENSG00000148204	CRB2	Crumbs 2, cell polarity complex component
Chr08	*Trans*	ENSGACG00000006901	ENSG00000136999	NOV	Nephroblastoma overexpressed
Chr09	*Cis*	ENSGACG00000019842	ENSG00000128272	ATF4	Activating transcription factor 4
Chr09	*Cis*	ENSGACG00000019868	ENSG00000103423	DNAJA3	DnaJ heat shock protein family (Hsp40) member A3
Chr09	Hotspot	ENSGACG00000019898	ENSG00000162961	DPY30	Dpy-30 histone methyltransferase complex regulatory subunit
Chr09	Hotspot	ENSGACG00000019915	ENSG00000132664	POLR3F	Polymerase (RNA) III (DNA directed) polypeptide F, 39 kDa
Chr09	Hotspot	ENSGACG00000020002	ENSG00000112658	SRF	Serum response factor
Chr09	Hotspot	ENSGACG00000019873	ENSG00000011243	AKAP8L	A-kinase anchoring protein 8 like
Chr12a	*Cis*	ENSGACG00000000816	ENSG00000126767	ELK1	ELK1, member of ETS oncogene family
Chr12a	Hotspot	ENSGACG00000000295	ENSG00000146109	ABT1	Activator of basal transcription 1
Chr12a	Hotspot	ENSGACG00000000248	ENSG00000106785	TRIM14	Tripartite motif containing 14
Chr12a	*Trans*	ENSGACG00000019625	ENSG00000164134	NAA15	N(α)-acetyltransferase 15, NatA auxiliary subunit
Chr12a	*Trans*	ENSGACG00000001088	ENSG00000111581	NUP107	Nucleoporin 107 kDa
Chr12b	*Cis*	ENSGACG00000006074	ENSG00000185513	L3MBTL1	L(3)mbt-like
Chr12b	*Cis*	ENSGACG00000004938	ENSG00000012504	NR1H4	Nuclear receptor subfamily 1, group h, member 4
Chr12b	Hotspot	ENSGACG00000011155	ENSG00000101017	CD40	CD40 molecule, TNF receptor superfamily member 5
Chr12b	Hotspot	ENSGACG00000010943	ENSG00000110925	CSRNP2	Cysteine-serine-rich nuclear protein 2
Chr12b	Hotspot	ENSGACG00000011240	ENSG00000163349	HIPK1	Homeodomain interacting protein kinase 1
Chr12b	Hotspot	ENSGACG00000011086	ENSG00000101096	NFATC2	Nuclear factor of activated T-cells, cytoplasmic, calcineurin-dependent 2
Chr12b	Hotspot	ENSGACG00000010788	ENSG00000123358	NR4A1	Nuclear receptor subfamily 4, group A, member 1
Chr12b	Hotspot	ENSGACG00000010925	ENSG00000184271	POU6F1	POU class 6 homeobox 1
Chr12b	Hotspot	ENSGACG00000010838	ENSG00000181852	RNF41	Ring finger protein 41, E3 ubiquitin protein ligase
Chr12b	Hotspot	ENSGACG00000011124	ENSG00000101115	SALL4	Spalt-like transcription factor 4
Chr12b	Hotspot	ENSGACG00000011135	ENSG00000182463	TSHZ2	Teashirt zinc finger homeobox 2
Chr12b	Hotspot	ENSGACG00000010929	ENSG00000135457	TFCP2	Transcription factor CP2
Chr12b	Hotspot	ENSGACG00000011187	ENSG00000204859	ZBTB48	Zinc finger and BTB domain containing 48
Chr12b	Hotspot	ENSGACG00000011128	ENSG00000020256	ZFP64	Zinc finger protein 64
Chr12b	Hotspot	ENSGACG00000010636	ENSG00000126895	AVPR2	Arginine vasopressin receptor 2
Chr12b	Hotspot	ENSGACG00000011168	ENSG00000171680	PLEKHG5	Pleckstrin homology and RhoGEF domain containing G5
Chr12b	Hotspot	ENSGACG00000011023	ENSG00000134242	PTPN22	Protein tyrosine phosphatase, nonreceptor type 22
Chr12b	*Trans*	ENSGACG00000011682	ENSG00000162761	LIMX1A	LIM homeobox transcription factor 1, α
Chr12c	*Cis*	ENSGACG00000013344	ENSG00000101997	CCDC22	Coiled-coil domain containing 22
Chr12c	*Cis*	ENSGACG00000013103	ENSG00000196924	FLNA	Filamin A, α
Chr12c	*Cis*	ENSGACG00000005361	ENSG00000116670	MAD2L2	Mitotic spindle assembly checkpoint protein MAD2B
Chr12c	*Cis*/hotspot	ENSGACG00000004839	ENSG00000188157	AGRN	Agrin
Chr12c	Hotspot	ENSGACG00000004256	ENSG00000101126	ADNP	Activity-dependent neuroprotector homeobox
Chr12c	Hotspot	ENSGACG00000004544	ENSG00000009307	CSDE1	Cold shock domain containing E1, RNA-binding
Chr12c	Hotspot	ENSGACG00000004732	ENSG00000101412	E2F1	E2F transcription factor 1
Chr12c	Hotspot	ENSGACG00000004740	ENSG00000078747	ITCH	Itchy E3 ubiquitin protein ligase
Chr12c	Hotspot	ENSGACG00000004213	ENSG00000197780	TAF13	TAF13 RNA Polymerase II, TATA box binding protein (TBP)-associated factor, 18 kDa
Chr12c	Hotspot	ENSGACG00000004773	ENSG00000122691	TWIST2	Twist homolog 2
Chr12c	Hotspot	ENSGACG00000004763	ENSG00000111424	VDR	Vitamin D (1,25- dihydroxyvitamin D3) receptor
Chr12c	Hotspot	ENSGACG00000004734	ENSG00000131061	ZNF341	Zinc finger protein 341
Chr12c	Hotspot	ENSGACG00000004662	ENSG00000197114	ZGPAT	Zinc finger, CCCH-type with G patch domain
Chr12c	Hotspot	ENSGACG00000004338	ENSG00000088832	FKBP1A	FK506 Binding Protein 1A
Chr16	*Cis*	ENSGACG00000005831	ENSG00000153234	NR4A2	Nuclear receptor subfamily 4, group A, member 2
Chr16	*Trans*	ENSGACG00000012487	ENSG00000125845	BMP2	Bone morphogenetic protein 2

Human orthologs of stickleback genes were identified using BioMart. Location is as follows: “Hotspot”: annotated gene is in genomic region of hotspot; “*Cis*”: gene is *cis*-regulated by hotspot at chromosome-wide *P* < 0.01; “*Trans*”: gene is *trans*-regulated by hotspot at genome-wide *P* < 0.021. Chr, chromosome.

### Upstream regulator and functional interaction analyses

When examining all 405 genes with *trans*-eQTL significant at genome-wide *P* < 0.021, 79 significantly enriched upstream regulators were identified using IPA (Table S8). In total, these regulators had 208 of the genes in the dataset as known targets. Hepatocyte nuclear factor 4α (HNF4A) was identified as a particularly important regulator (*P* = 9.3 × 10^−8^), with 70 (33.7%) of these genes as downstream targets. Other highly enriched regulatory factors included one cut homeobox 1 (ONECUT1; *P* = 3.2 × 10^−5^; 16 target genes), Nuclear Receptor Subfamily 4 Group A Member 1 (NR4A1; *P* = 2.0 × 10^−4^; 11 genes), Signal Transducer And Activator Of Transcription 5B (STAT5B; *P* = 5.5 × 10^−4^; 10 genes), Krüppel-like factor 3 (KLF3; *P* = 8.7 × 10^−4^; 15 genes), estrogen receptor 1 (ESR1; *P* = 1.8 × 10^−3^; 37 genes); Hepatocyte nuclear factor 1α (HNF1A; *P* = 1.9 × 10^−3^; 17 genes); CAMP Responsive Element Binding Protein 1 (CREB1; *P* = 3.2 × 10^−3^; 19 genes), and myc proto-oncogene protein (MYC; *P* = 3.4 × 10^−3^; 30 genes). The full list of 79 significant upstream regulators is in Table S8.

To identify upstream regulators that could be contributing to the 10 eQTL hotspots, we further examined all genes that had *trans*-eQTL mapping to the hotspots at genome-wide *P* < 0.057 (1120 genes). One hundred and ninety-two different enriched upstream regulators were identified for these genes (Table S8). For genes with *trans*-eQTL mapping to the Chr4b, Chr6, Chr12a, and Chr12c hotspots, HNF4A remained an important regulator. Only five of the identified upstream regulators were physically located within a hotspot (*NFKB1*, Chr4a; *SOX3*, Chr4a; *SRF*, Chr9; *NFATC2*, Chr12b; and *NR4A1*, Chr12b). Five had significant *cis*- or *trans*-eQTL mapping to a hotspot (*IRF2*, Chr4a *trans*; *NCOA4*, Chr6 *cis*; *HIF1A*, Chr6 *trans*; *JUP*, Chr7 *trans*; and ELK1, Chr12a *cis*). None of these 10 hotspot-associated regulatory proteins were identified as significant upstream regulators for the sets of genes with *trans*-eQTL mapping to the same hotspot; in other words, their presence did not appear to be causative of the observed hotspots.

**Figure 2:**
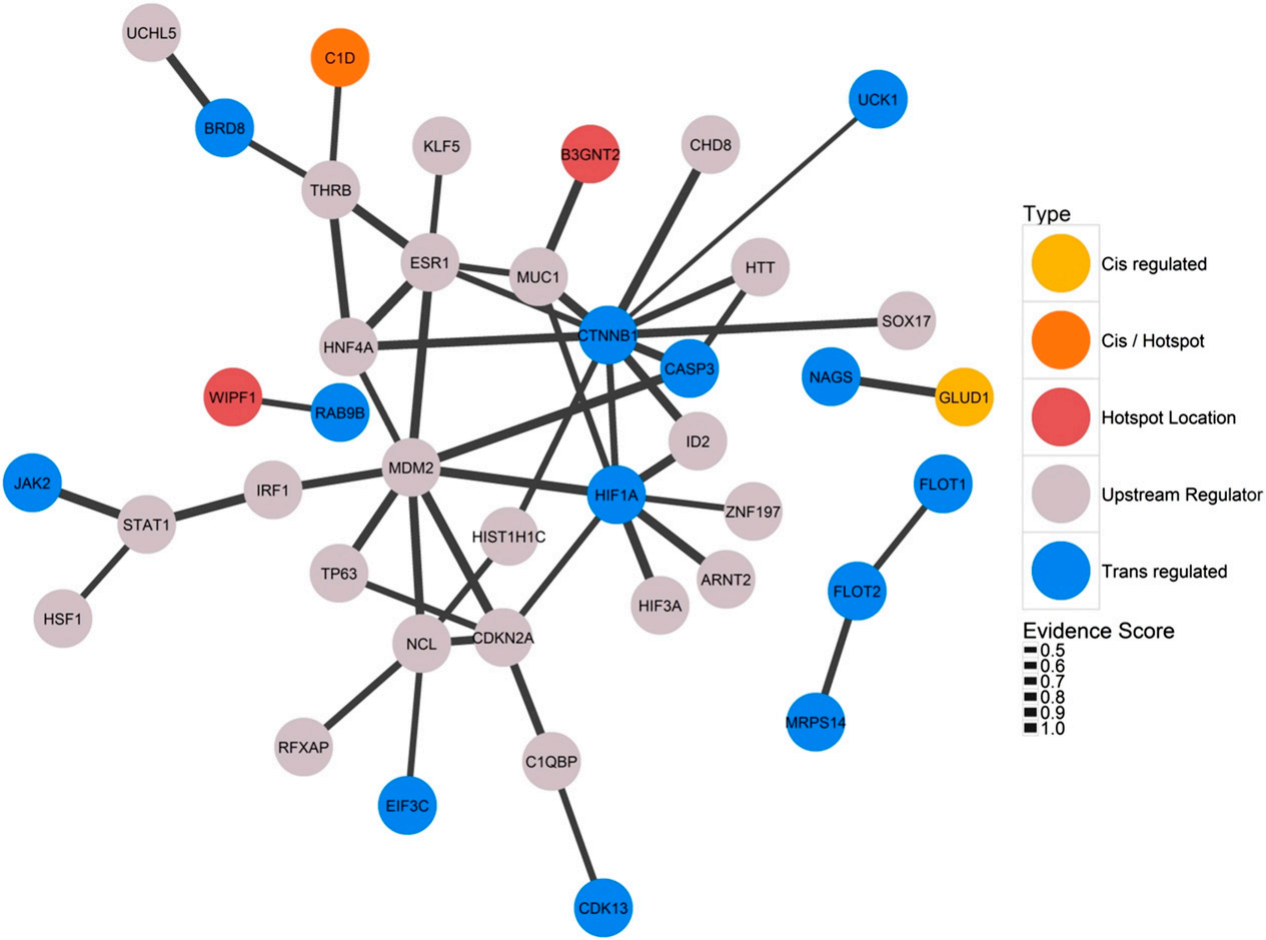
Networks of known protein–protein interactions inferred by String 10 for proteins associated with the Chr6 hotspot. “Upstream Regulator”: significantly enriched upstream regulator identified when examining genes *trans*-regulated by the hotspot using ingenuity pathway analysis; “Hotspot Location”: protein is coded by a gene physically located in the hotspot; “Trans regulated”: protein is *trans*-regulated by an eQTL mapping to the hotspot and significant at genome-wide *P* < 0.021; Cis/Hotspot: both present in and significantly *cis*-regulated by the hotspot. Interactions not involving an identified upstream regulator are not shown.

When the enriched upstream regulators, genes with *cis*-eQTL mapping to a hotspot at chromosome-wide *P* < 0.01, and genes with *trans*-eQTL mapping to a hotspot at genome-wide *P* < 0.021 were examined in STRING, multiple protein–protein interactions were found (Figure 2, Figure S4). In particular, for the Chr6 hotspot we found a complex interaction network that included eight molecules *trans*-regulated by this hotspot (in order of connectivity: CTNNB1, HIF1A, CASP3, BRD8, CDK13, EIF3C, JAK2, and UCK1), two molecules *cis*-regulated by the hotspot (C1D and B3GNT2), and multiple molecules inferred as important upstream regulators by IPA (Figure 2).

## Discussion

In this study, we identified regions of the genome underlying variation in gene expression in a population of threespine stickleback from northern Europe. We used a genotyping-by-sequencing approach to generate an improved linkage map, and applied interval mapping to identify eQTL. Our new map was independent of that recently constructed by Glazer *et al.* (2015), and the congruent placement of scaffolds between the two maps confirms the reliability of these new genome assemblies. Our map covered a substantially larger distance in centimorgans than those of Roesti *et al.* (2013) and Glazer *et al.* (2015), probably due to differences in experimental design. Nevertheless, for our Baltic Sea population, we observe very similar patterns of recombination rate variation across and between chromosomes as found by Roesti *et al.* (2013) for freshwater sticklebacks from central Europe and Glazer *et al.* (2015) for marine–freshwater crosses from western North America (Figure S1). Thus, the large-scale pattern of recombination rate variation across the genome may impose, and/or be under, similar evolutionary constraints throughout the range of the species.

Using a chromosome-wide significance threshold for *cis*-regulatory loci and a genome-wide threshold for *trans*-loci, we identified eQTL for just over a quarter of the 10,332 expression traits examined. Because at least 74% of these expression traits exhibit significant heritable variation (Leder *et al.* 2015), and gene expression is commonly regulated by multiple eQTL, we expect that a much larger number of underlying eQTL remain undetected due to low statistical power. Despite expectations that *trans*-regulatory regions might be under purifying selection due to their potentially pleiotropic effect, and that the effect of *trans*-eQTL on expression will be weaker than that of *cis*-eQTL, we found many cases where gene expression was influenced by regulatory variation in *trans* but not in *cis*. This suggests that a frequently-used approach of detecting local selection by examining patterns of differentiation at markers linked to genes that are adaptive candidates (*e.g.*, DeFaveri *et al.* 2011, Shimada *et al.* 2011) may fail to identify such selection as it is acting to change gene expression via *trans*-regulatory regions. We did not observe any difference in additive *vs.* dominance variance underlying genes found to be regulated in *cis* *vs.* those regulated in *trans*. However, this may again be due to low statistical power to detect many of the underlying eQTL: genes are expected to be influenced by a large number of eQTL, meaning that the observed heritable variation is generated by a combination of additively- and nonadditively-acting regulatory regions.

The *trans*-eQTL that we detected were not randomly distributed across the genome but instead clustered into multiple eQTL hotspots. This observation is a ubiquitous feature of eQTL studies and is thought to indicate the existence of “master regulators” acting in *trans* to influence many genes. However, apparent eQTL hotspots may also arise as a statistical artifact as a result of many false positive QTL when testing thousands of expression traits in combination with spurious correlation between these traits due to uncorrected experimental factors (Wang *et al.* 2007; Breitling *et al.* 2008). Disentangling gene expression correlation that is due to common underlying regulatory architecture from that caused by experimental artifacts is a difficult analytical problem that we are unable to fully address here (Joo *et al.* 2014). Therefore, we caution that these hotspots should be verified using other stickleback populations and different approaches.

The parents for this study came from a genetically diverse marine population of threespine stickleback (DeFaveri *et al.* 2013). Local adaptation of threespine sticklebacks to freshwater has been demonstrated to arise, at least partly, from selection on standing genetic variation in the marine environment. Further, QTL underlying morphological divergence between marine and freshwater populations have been demonstrated to have pleiotropic effects (Rogers *et al.* 2012; Miller *et al.* 2014), and frequently colocalize with regions of the genome found to be under parallel selection among independent freshwater colonizations. One way in which these regions could exert such pleiotropic effects is by harboring loci that influence the expression of many genes, *i.e.*, eQTL hotspots. However, only one of the *trans*-eQTL hotspots found in this study (Chr12a) overlapped with genomic regions repeatedly found to be associated with marine/freshwater divergence by Hohenlohe *et al.* (2010), Jones *et al.* (2012), or Terekhanova *et al.* (2014).

Nevertheless, several studies indicate that adaptation to novel aquatic environments may also involve parts of the genome outside these large target regions (DeFaveri *et al.* 2011; Leinonen *et al.* 2012; Ellis *et al.* 2015; Erickson *et al.* 2016; Ferchaud and Hansen 2016). The QTL underlying physiological adaptations to different aquatic environments in sticklebacks have not been well characterized. Recently, Kusakabe *et al.* (2016) identified a significant QTL associated with salinity tolerance (indicated by gill sodium plasma levels) on chromosome 16, which overlaps our Chr16 *trans*-eQTL hotspot. Interestingly, this also appears to overlap with a chromosome 16 QTL underlying gill raker morphology identified by Glazer *et al.* (2015). Based on transcription levels, Kusakabe *et al.* (2016) identified 10 candidate causal genes at the QTL location; we found *cis*-regulatory variation for four of these genes (*CLN5, IGFBP5, RABL3*, and *NDUFA10*) and a fifth (*GDP-like*) had a *trans*-eQTL mapping to the Chr4a hotspot. However, Kusakabe *et al.* (2016) did not investigate genes located elsewhere on the genome that may be *trans*-regulated by this chromosome 16 QTL. Our results also show that all genes with *trans*-eQTL mapping to the Chr16 hotspot exhibit a plastic response to the temperature treatment. Thus, the Chr 16 eQTL hotspot may be involved in physiological adjustment to several environmental variables.

Identifying eQTL directly implicated in local adaptation in sticklebacks was not our experimental aim, and it is possible that regulatory hotspots acting in tissues or life stages that we did not examine have a role in stickleback adaptive radiation. In general, it is difficult to predict in which tissues, or at which life stages, gene expression variation gives rise to observed adaptive differences. We examined transcription in the liver, an easily accessible, metabolically active tissue. The liver expresses many genes with potential roles in the physiological adaptation to different aquatic environments, including hormone receptors and genes involved in osmoregulation, energy homeostasis, and response to hypoxia. Further, many eQTL identified in this study may be common to other tissues. In general, the extent to which eQTL are shared among tissues remains unclear, due to the need for very large sample sizes and the limitations of the statistical methodologies available to address this question (The GTEx Consortium 2015). In particular, variation in gene expression levels among tissues means that the power to detect underlying eQTL also varies among tissues. Although studies have suggested that up to 70% of genes may have common underlying eQTL across tissues (Nica *et al.* 2011), there is also some evidence that *trans*-eQTL hotspots in particular may act in a tissue-specific manner (Grundberg *et al.* 2012). Thus, replication of this study in a greater range of tissues, and at different life stages, would shed more light on the regulatory genetic architecture underlying the parallel changes observed when marine sticklebacks independently colonize freshwater.

To investigate the potential genetic mechanisms generating the nine observed eQTL hotspots, we searched for associated loci with known transcriptional regulatory functions, and performed upstream regulator analysis for the genes with eQTL in the hotspots. Although the pathways regulating transcription are still poorly characterized for most genes, particularly in nonmammalian species, these analyses can provide useful preliminary information. We found no evidence that eQTL hotspots were due to the presence of a single “master” regulatory locus, or a cluster of regulatory genes, at the hotspot locations. Although many genes with roles in transcriptional regulation were present in, or regulated by, hotspots, finding such genes is not unexpected: ∼18% of the human orthologs of BROAD stickleback genes are annotated with the GO terms that we used to identify transcriptional regulators. It is also possible that the regulatory elements generating such hotspots are not annotated coding genes: microRNAs and long noncoding RNAs are potentially important *trans* regulators (Vance and Ponting 2014) and not yet well characterized across the stickleback genome.

Our results suggest that, alternatively, these hotspots may be generated by a complex interaction of multiple transcription regulators. Several well-characterized regulatory proteins were identified as important upstream regulators for genes with *trans*-eQTL mapping to the hotspots. Unsurprisingly, these included three genes—*HNF4A*, *ONECUT1* and *HNF1A*—known to be master transcriptional regulators in the mammalian liver (Odom *et al.* 2004). HNF4A and ONECUT1 were identified as particularly strongly enriched upstream regulators when examining all genes with a *trans*-eQTL at genome-wide *P* < 0.021 (Table S8), and were also found to be enriched when examining the subsets of genes with *trans*-eQTL mapping to the hotspots on chromosome 4, 6, and 12 (Table S8). None of the three genes were physically located in any hotspot, and we were unable to identify significant eQTL underlying variation in their expression (*ONECUT1* was not on the microarray). However, we note that *HNF4A* is < 300 kb from hotspot Chr12b. These regulators likely act through direct and indirect interactions with other proteins to regulate transcription. Interacting molecules that are especially of interest in respect to hotspot locations are hypoxia inducible factor 1α and catenin β-1 (*HIF1A* and *CTNNB1*, *trans*-regulated by the Chr6 hotspot, Figure 2), histone deacetylase 3 (*HDAC3*, located in the Chr4b hotspot, Figure S4), and vitamin D receptor (*VDR*, located in the Chr12c hotspot, Figure S4).

The protein HIF1A has previously been investigated as a selective target of local adaptation in fish. It is part of a transcriptional complex (HIF) that alters the expression of numerous genes in many tissues in response to low oxygen conditions (Nikinmaa and Rees 2005, Liu *et al.* 2013). It is also involved in temperature adaptation in fish (Rissanen *et al.* 2006; Liu *et al.* 2013). Thus, HIF1A is of relevance when fish colonize aquatic environments with differing oxygen regimens, for example benthic *vs.* limnetic habitats or different areas of the Baltic Sea. Rytkönen *et al.* (2007) found no association between variation in the *HIF1A* coding region and adaptation to hypoxic conditions across various fish species, and markers linked to *HIF1A* do not appear be under directional selection among Baltic Sea stickleback populations (Shimada *et al.* 2011); however, the gene was recently found to be under positive selection in high-altitude loach lineages (Wang *et al.* 2015). *HIF1A* is known to be transcriptionally regulated in fish (Liu *et al.* 2013), and our identification of a *trans*-eQTL for *HIF1A* demonstrates that regulatory variation for this gene is present in Baltic Sea sticklebacks and could be an alternative, unexamined, target of selection. The proteins HNF4A, CNNB1, and HDAC3 are also involved in the hypoxia response (Xu *et al.* 2011; Wang *et al.* 2015).

In conclusion, we have performed the first genome-wide characterization of the regulatory architecture of gene expression in *G. aculeatus*. We found that variation in gene expression was influenced by polymorphism in both *cis*-acting and *trans*-acting regulatory regions. *Trans*-acting eQTLS clustered into hotspots. In general, these hotspots did not colocate with regions of the genome known to be associated with parallel adaptive divergence among marine and freshwater threespine sticklebacks. However, one hotspot overlapped with a known QTL underlying salinity tolerance, a locally adaptive trait. Hotspot locations appeared to be mediated by complex interactions among regulator molecules rather than the presence of few “master regulators.” Our broad-scale study suggests many avenues for finer-scale investigation of the role of transcriptional regulation in stickleback evolution.

## Supplementary Material

Supplemental material is available online at www.g3journal.org/lookup/suppl/doi:10.1534/g3.116.033241/-/DC1.
