# Supplementary figures and images for: Regulatory Architecture of Gene Expression Variation in the Threespine Stickleback Gasterosteus aculeatus
Source: G3 (Bethesda). 2016 Nov 10;7(1):165–78. doi: 10.1534/g3.116.033241 (PMC5217106; doi:10.1534/g3.116.033241)

Figure S1

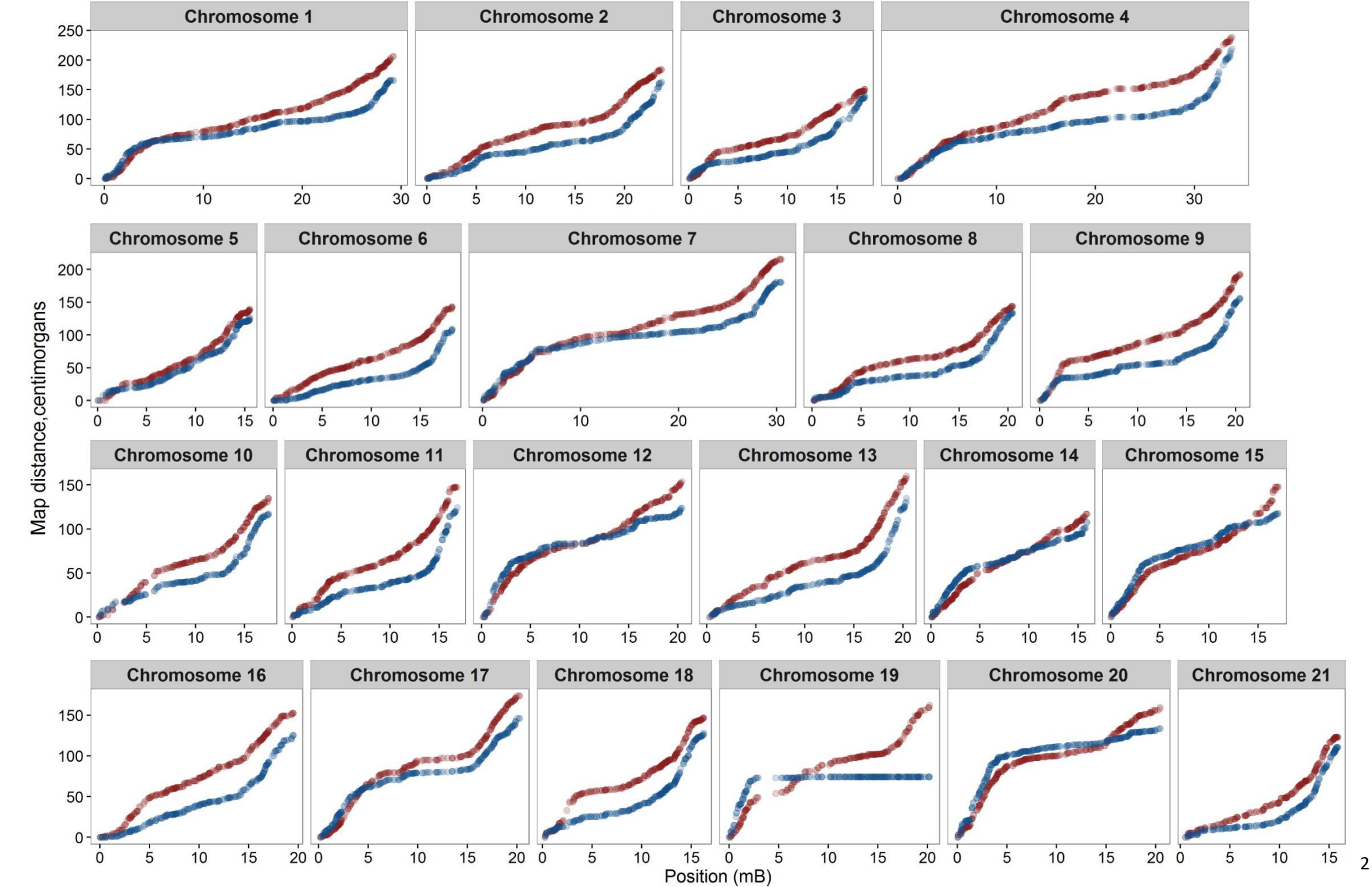

Supplement: Supplementary file 1 [file 165FigureS1.pdf]

Figure S2

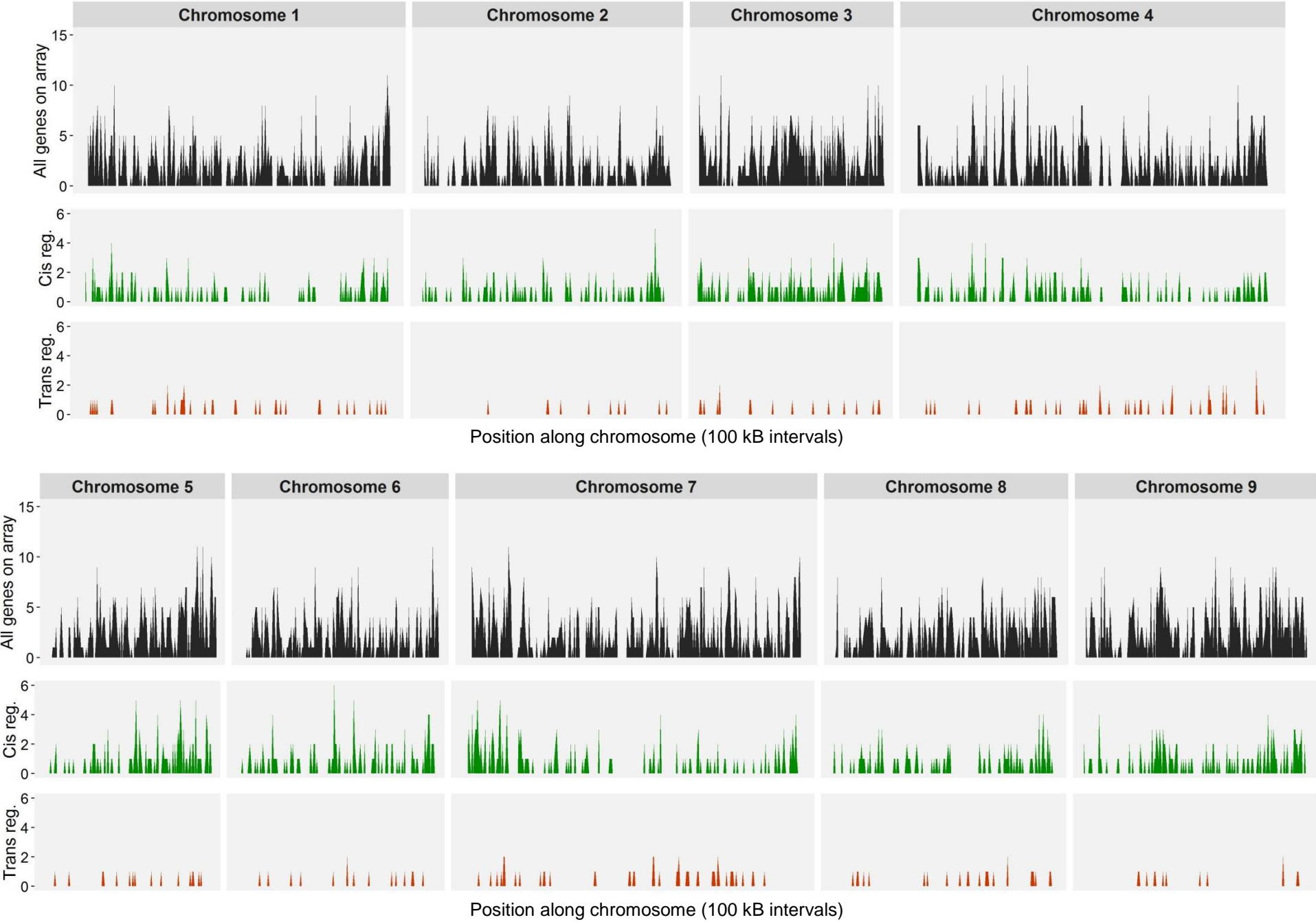

Figure S2 (cont.)

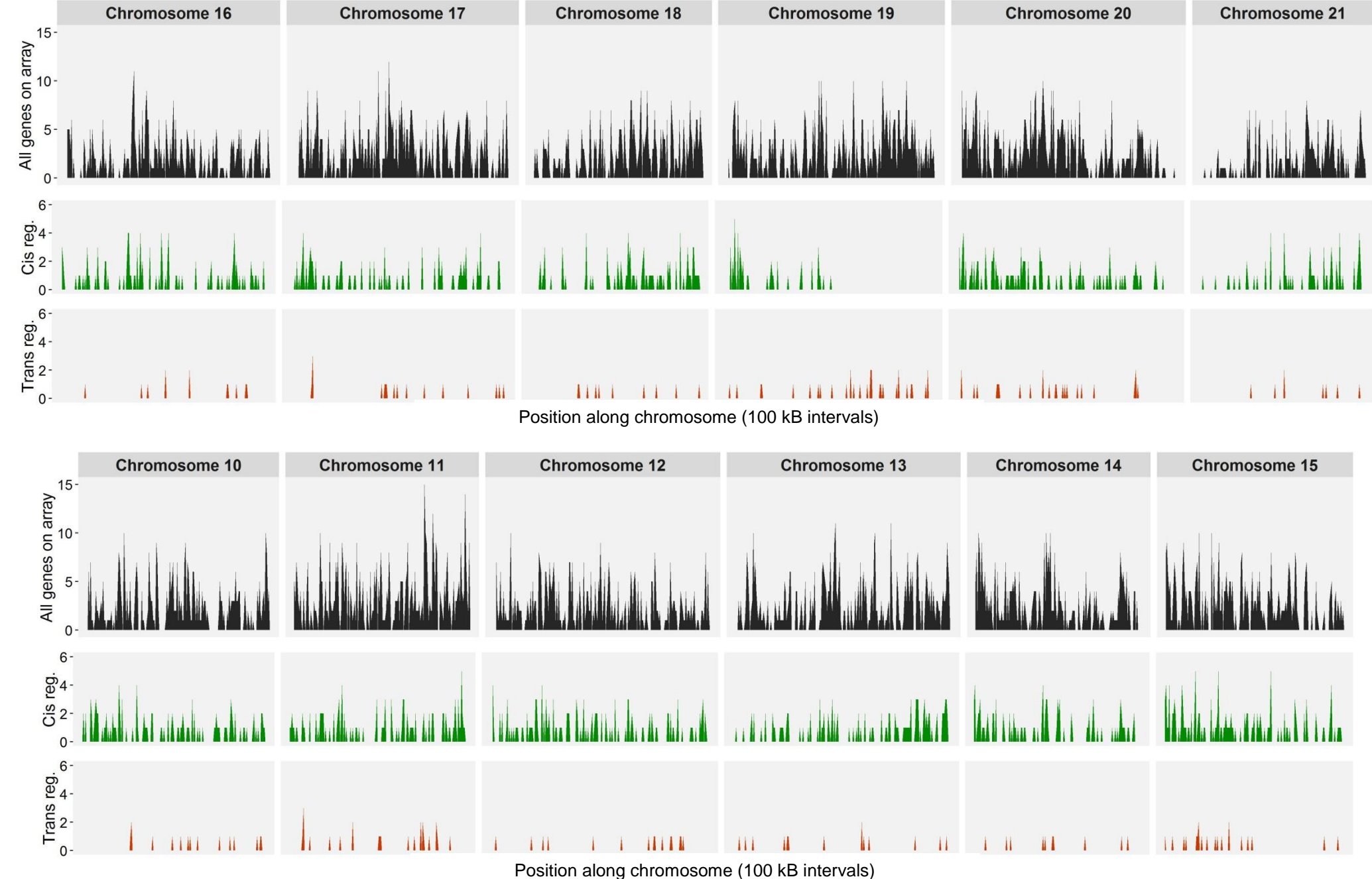

Supplement: Supplementary file 2 [file 165FigureS2.pdf]

Figure S3

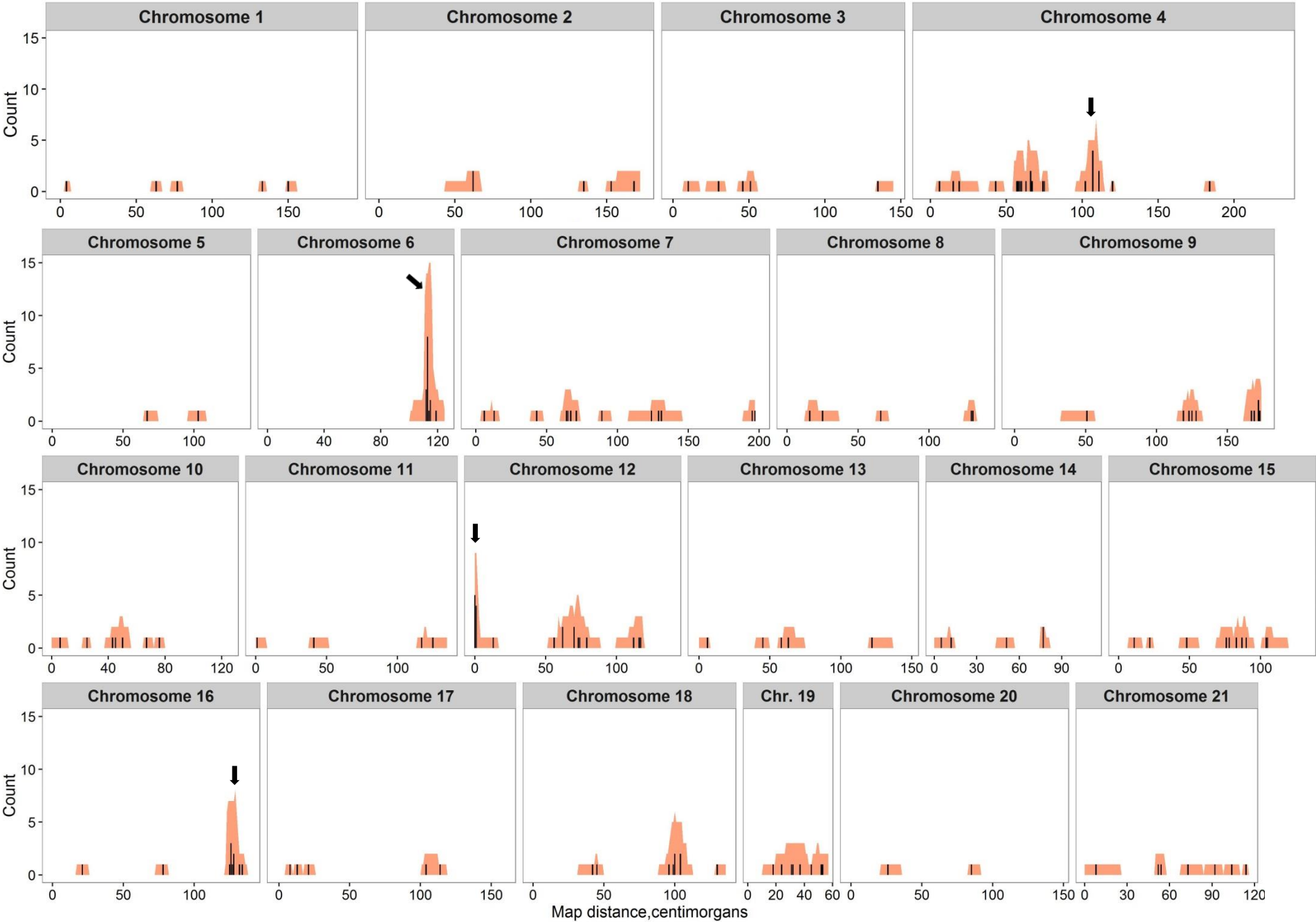

Supplement: Supplementary file 3 [file 165FigureS3.pdf]

Figure S4

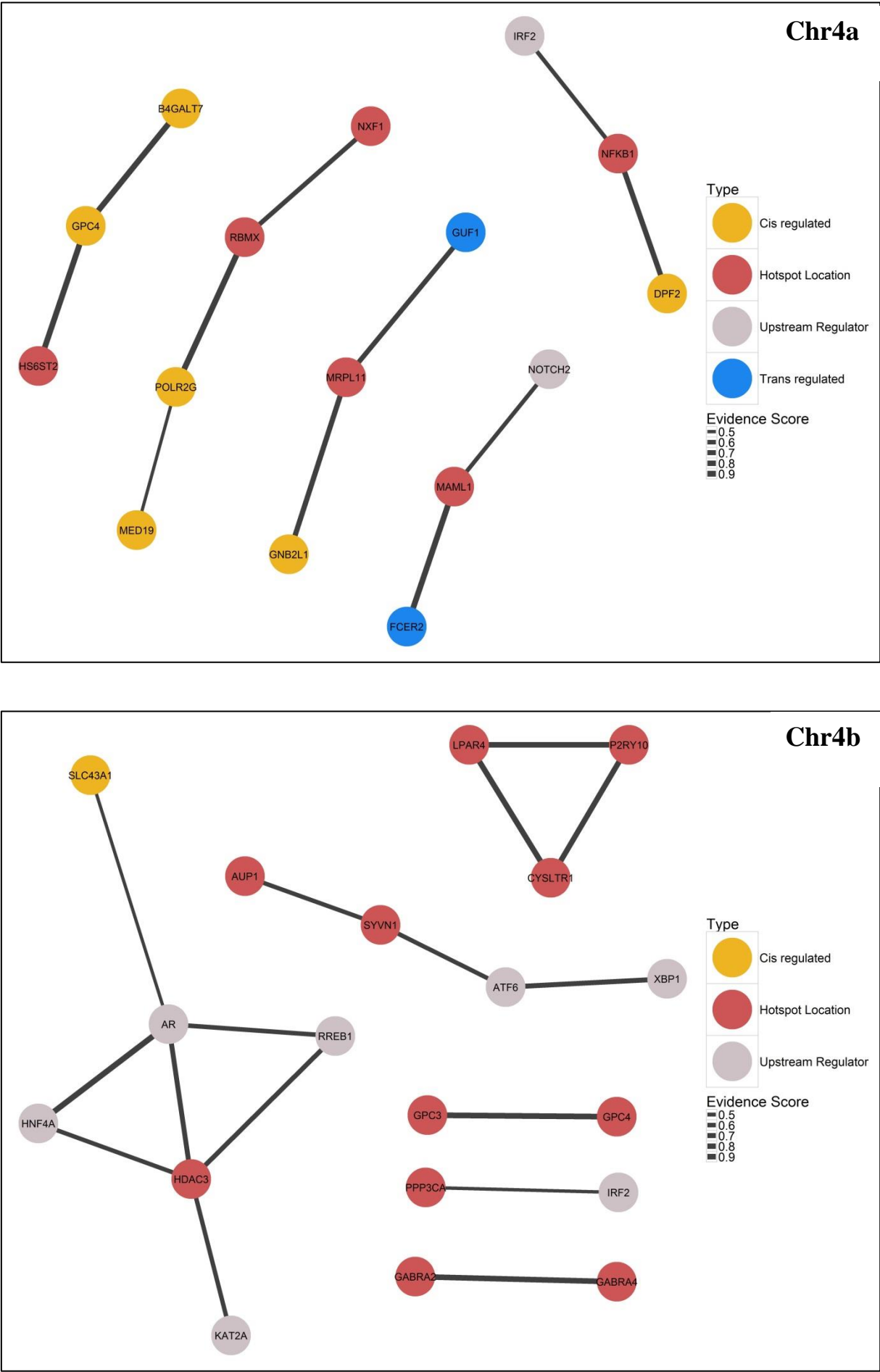

Figure S4 (cont)

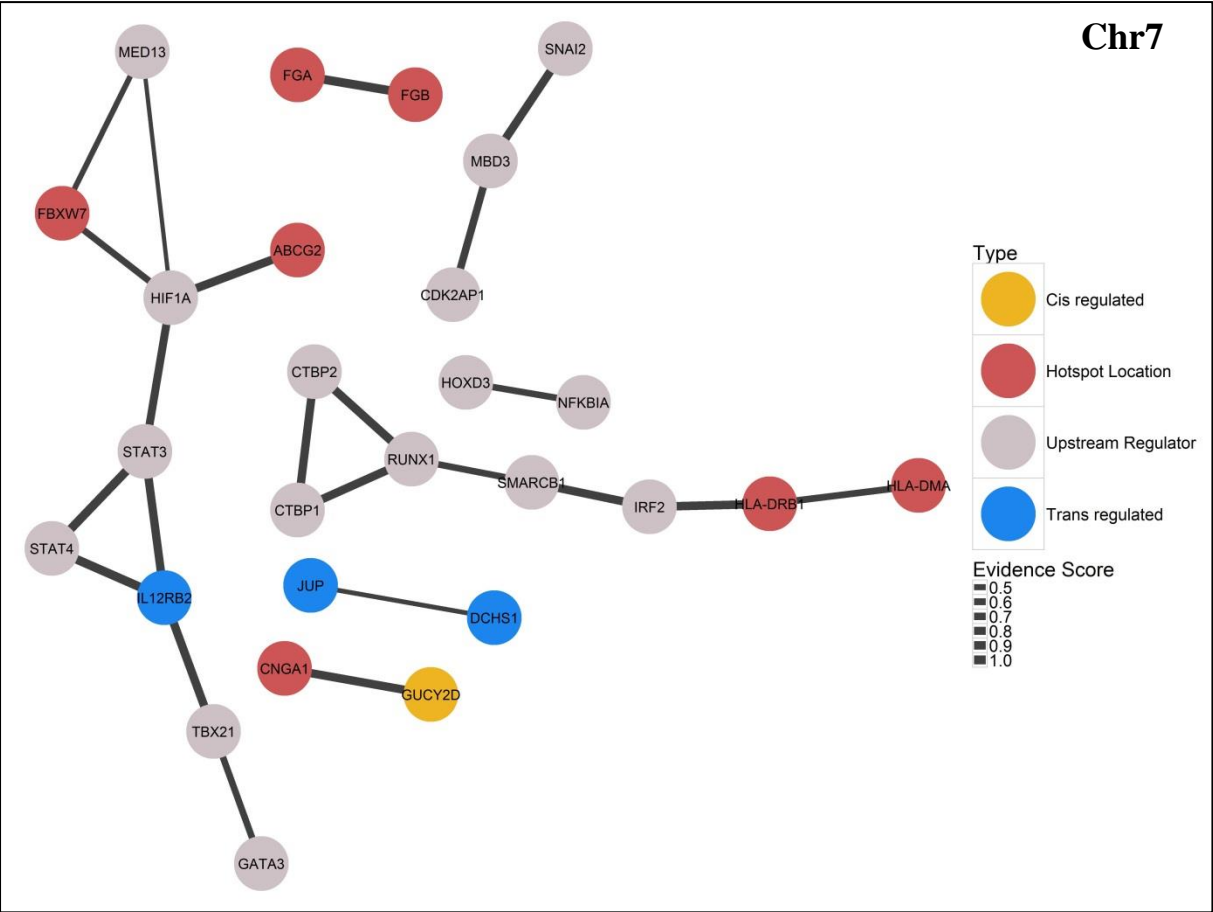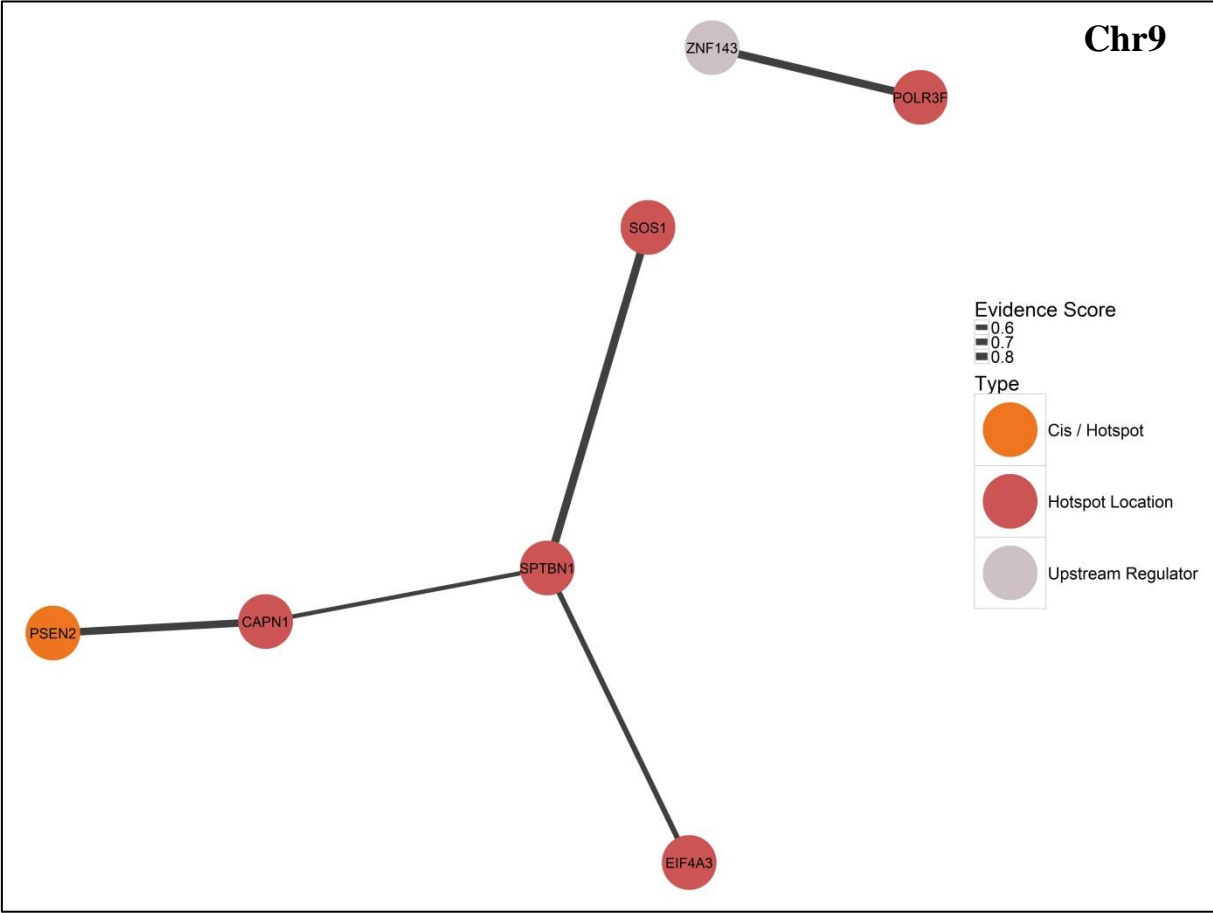

Figure S4 (cont.)

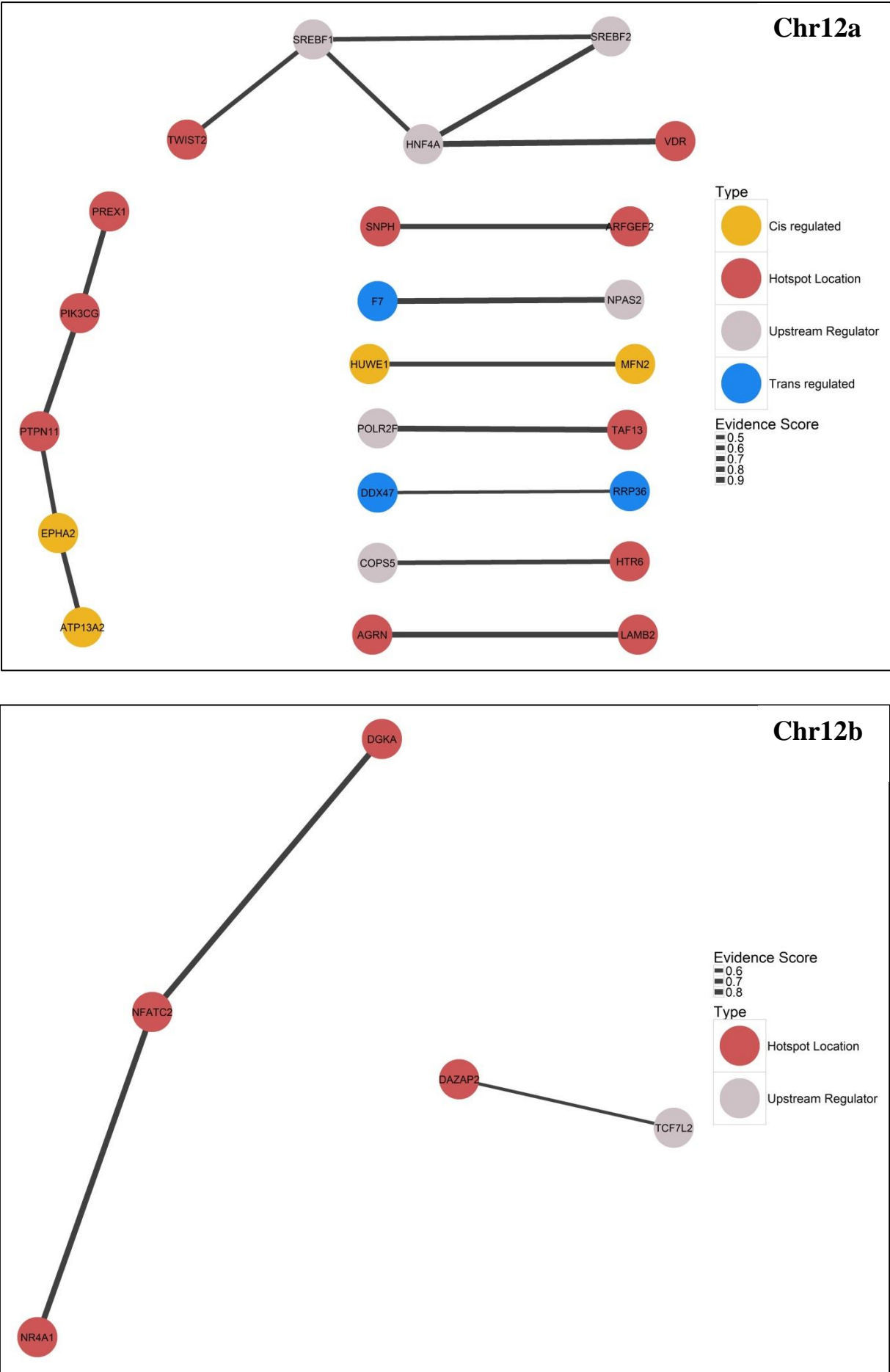

Figure S4 (cont)

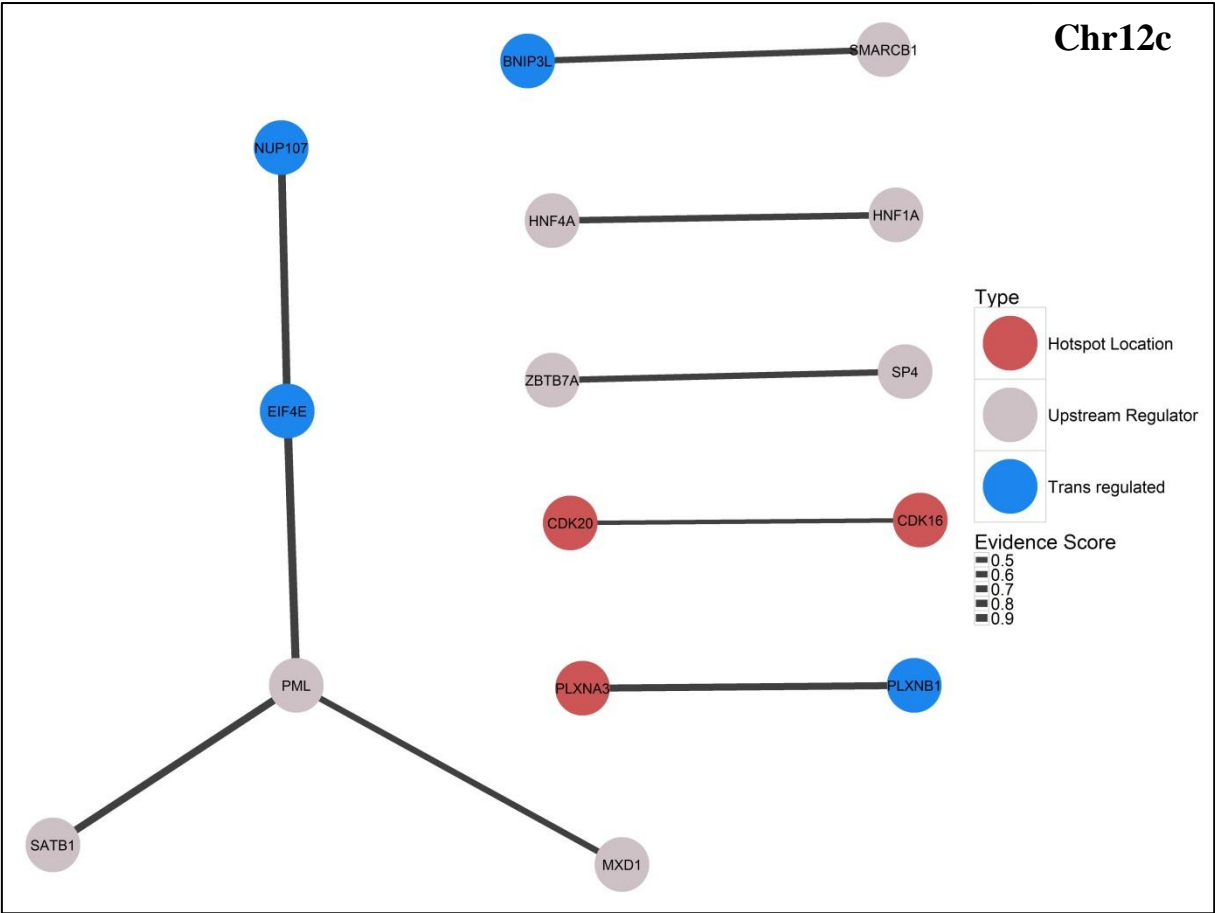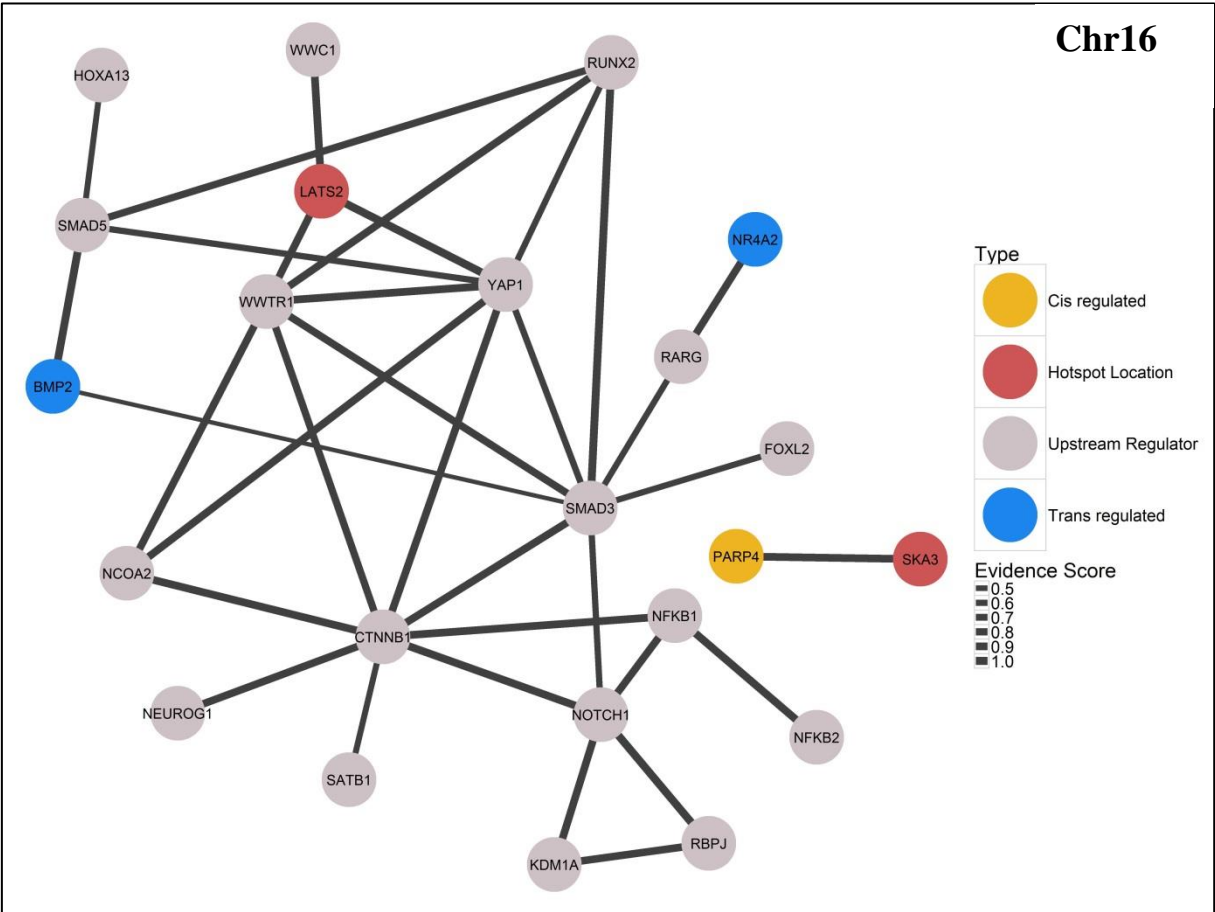

Supplement: Supplementary file 4 [file 165FigureS4.pdf]
