# Supplementary material for: Regulatory Architecture of Gene Expression Variation in the Threespine Stickleback Gasterosteus aculeatus
Source: G3 (Bethesda). 2016 Nov 10;7(1):165–78. doi: 10.1534/g3.116.033241 (PMC5217106; doi:10.1534/g3.116.033241)
Supplement: Supplementary file 11 [file 165FileS7.pdf]

REGULATORY ARCHITECTURE OF GENE EXPRESSION VARIATION IN THE THREESPINE STICKLEBACK, *GASTEROSTEUS ACULEATUS.*

Legends for Supplementary Tables and Files.

Table S1: Raw and aligned read coverage for individual sticklebacks included in the analysis.

Table S2: Stickleback linkage map generated by this study. SNP: SNP marker name, which indicates group/scaffold and position on the Broad S1 stickleback genome; Recoded_chromosome, Recoded_position: location of SNP on the recoded map; All_cM: location of SNP in Kosambi cM, averaged over sires and dams; Dam_cM: location of SNP estimated from dams alone; Sire_cM: location of SNP estimated from sires alone. SNPs included in the genetic map provided to QTLMap are indicated by 'Y' in the first column.

Table S3: Placement and orientation of scaffolds in modified *G. aculeatus* genome build. Scaffolds are listed in the order in which they are placed along the chromosome. 'Build' indicates whether scaffold placement and orientation is based on the original BROAD S1 build ('Original'), Roesti *et al.* (2012) ('Roesti'), or new for this study ('New'). 'Glazer differences' indicate differences in scaffold placement and orientation between this build and that of Glazer *et al.* (2015).

Table S4: Results of eQTL analysis. Column headings are as follows:

Transcript**:** Ensembl ID of transcript assayed by microarray.

eQTL chrom, eQTL position (cM): location of QTL on the genetic map.

LRT: likelihood ratio test value for QTL.

eQTL.CW.sig: chromosome-wide significance of QTL, estimated empirically from distribution of LRT values over 5000 permuted datasets. Genome-wide significance = 21x chromosome-wide significance. * $p < 0.01$; ** $p < 0.005$; *** $p < 0.0027$; **** $p < 0.001$; ***** $p < 0.0005$; ****** $p < 0.0001$.

# Families**:** number of half-sib families where QTL effect is significant ($p < 0.05$ based on student t test, calculated by QTLMap).

Weighted.mean.effect.size: Weighted mean QTL allele substitution effect, in phenotypic standard deviations. Calculated by QTLMap over all sires with a significant QTL effect, as the mean of ((2*absolute sire QTL effect)/experiment-wide SD).

Cis/Trans: location of eQTL in relation to regulated gene: *cis*, *trans* or unknown

Outlying.region?: Does the 95% confidence interval of eQTL location overlap genomic regions identified as outliers in Hohenlohe et al. (2010), Jones et al. (2012), or Terekhanova et al. (2014)?

Flanking.SNP.1, Flanking.SNP.2: SNP markers immediately flanking eQTL position

Flanking.SNP.1.position, Flanking.SNP.2.position: Base-pair position of the flanking SNPs on the recoded genome build.

eQTL.lower.95%CI; eQTL.upper.95%CI**:** lower and upper 95% confidence intervals of QTL location, position on the chromosome in cM.

Lower.95%CI.SNP, Upper.95%CI.SNP: Closest SNP markers to lower and upper 95% confidence intervals of QTL location.

Lower.95%CI.position, Upper.95%CI.position: Base-pair position of the latter SNPs on the recoded genome build.

Gene: Ensembl ID of gene generating transcript.

Gene.chrom, Gene.start.position, Gene.end.position: location of gene on the recoded genome build, based on the BROAD S1.77 stickleback genome annotation.

Gene.name: name of gene in the BROAD S1.77 stickleback genome annotation.

Human.HGNC.symbol: HUGO Gene Nomenclature Committee symbol for the orthologous human gene.

Sig.VA; Sig.VD: 'YES' indicates that the transcript was found to have significant underlying additive or dominance variance by Leder *et al.* (2015).

h^2; d^2: narrow-sense heritability and dominance variance of transcript expression, from Leder *et al.* 2015.

FDR.βEnv: FDR corrected probability that transcript expression was affected by the temperature treatment, from Leder et al. (2015).

Table S5: Original BROAD S1.77 annotation, and re-coded gene locations used for this study. **'**Array_transcript' shows Ensembl IDs for the transcripts assayed with the microarray. 'sv' in the 'Splice_variant' column indicates that more than one splice variant is assayed for a gene.

Table S6: Re-coded gene locations and location of eQTL hotspots ('Hotspot') in relation to outlying regions of the genome found in marine/freshwater comparisons by Hohenlohe *et al.* (2010), Jones *et al.* (2012) and Terekhanova *et al.* (2014), and outliers amongst Baltic Sea populations found by 'Significant_eQTL' indicates type (*cis/trans*) of significant eQTL associated with each transcript.

Table S7: Genes located in or with eQTL in eQTL hotspots that have GO terms related to transcriptional regulation. For completeness genes with trans eQTL significant at the chromosome-wide level but not at the genome-wide level (indicated by "Trans (non-significant)") are included.

Table S8: IPA identified upstream regulators for all human-annotated genes with *trans* eQTL significant at genome-wide p<0.021, and all human-annotated genes with *trans* eQTL significant at genome-wide p<0.054 and mapping to eQTL hotspots.

Files S1-S5: Input files for QTLMap.

File S6: Information about archived RAD reads and microarray results. Column headings are as follows:

Sample.id.PRJNA340327: Individual IDs for RAD reads archived in the NCBI Sequence Read Archive under BioProject ID PRJNA340327.

Sample.id.E-MTAB-3098: Individual IDs for microarray data archived in the ArrayExpress database under accession number E-MTAB-3098.

Treatment: Whether or not an individual received the temperature treatment. Entries in this column supercede those in E-MTAB-3098.
